# Supplementary material for: Single-cell AI-based detection and prognostic and predictive value of DNA mismatch repair deficiency in colorectal cancer
Source: Cell Rep Med. 2024 Sep 17;5(9):101727. doi: 10.1016/j.xcrm.2024.101727 (PMC11525017; doi:10.1016/j.xcrm.2024.101727)
Supplement: Document S1. Figures S1–S9 and Tables S1–S8 [file mmc1.pdf]

Cell Reports Medicine, Volume *5*

Supplemental information

Single-cell AI-based detection and prognostic and predictive value of DNA mismatch repair deficiency in colorectal cancer

Marta Nowak, Faiz Jabbar, Ann-Katrin Rodewald, Luciana Gneo, Tijana Tomasevic, Andrea Harkin, Tim Iveson, Mark Saunders, Rachel Kerr, Karin Oein, Noori Maka, Jennifer Hay, Joanne Edwards, Ian Tomlinson, Owen Sansom, Caroline Kelly, Francesco Pezzella, David Kerr, Alistair Easton, Enric Domingo, TransSCOT group, Viktor H. Koelzer, and David N. Church

Supplemental information to Nowak et al.

Single cell AI-based detection, prognostic and predictive value of DNA mismatch repair deficiency in colorectal cancer.

Supplementary material

Contents

Table S1. Details of training and test set used for the AIMMeR development in HALO digital image analysis software v3.3. ... 2

Table S2. Agreement between AI and pathologist review for classification of MMR status and protein loss ... 3

Table S3. Classification of MMR protein loss by AI and consensus pathology review by type of MMR loss ... 4

Table S4. Reasons for discordance between AI and consensus pathologist classification ... 5

Table S5. Reasons for discordance between individual pathologist classification ... 6

Table S6. Clinicopathological characteristics of SCOT trial by MMR status ... 7

Table S7. Univariable and multivariable analysis of recurrence-free interval in SCOT trial cohort according to clinicopathologic factors and MMR status ... 8

Table S8. Biomarker analyses performed and reported in this study ... 9

Figure S1. Nuclear segmentation by object class ... 10

Figure S2. Scatterplots showing correlation between epithelial MMR protein expression across cases 11

Figure S3. Scatterplots showing correlation between stromal MMR protein expression across cases .. 12

Figure S4. Calculation of AUROC for alternative methods for classification of MMR loss ... 13

Figure S5. Relationship between AI and consensus pathologist calls for combinations of MMR protein loss ... 16

Figure S6. Cases discordant between AI-based and pathologist classification ... 17

Figure S7. Tumor lymphocytic infiltrate and tumor/stroma ratio by MMR status ... 18

Table S1. Details of training and test set used for the AIMMeR development in HALO digital image analysis software v3.3. Related to STAR methods

**Training Set**		
**Class**	**Objects**	**Accuracy**
positive tumor cells	31,786	NA
negative tumor cells	16,607	NA
positive stroma	20,881	NA
negative stroma	13,030	NA
lymphocytes (positive and negative)	19,404	NA
strong background	22,356	NA
weak background	2,848	NA
sum	126,912	NA
**Test Set**		
**Class**	**Object used for test**	**Accuracy**
positive tumor cells	1796	0.95
negative tumor cells	1604	0.85
positive stroma	1004	0.93
negative stroma	868	0.89
lymphocytes (positive and negative)	2471	0.98
strong and weak background	1200	0.92
sum/overall accuracy	8,943	0.92

Table S2. Agreement between AI and pathologist review for classification of MMR status and protein loss Related to Figure 3.

Group	Comparison	Number of cases	Cohen kappa (95% CI)	Gwet AC1 (95% CI)
Cases with consensus pathologist review ± blinded individual pathologist review				
MMR status (retained vs lost)	AI vs pathologist consensus	685	0.79 (0.75–0.84)	0.87 (0.84–0.90)
	AI vs pathologist 1 (AE)	601*	0.82 (0.78–0.87)	0.89 (0.86–0.92)
	AI vs pathologist 2 (VK)	601*	0.79 (0.74–0.84)	0.85 (0.82–0.89)
	Pathologist 1 vs 2	601*	0.88 (0.84–0.92)	0.92 (0.89–0.94)
Individual MMR protein loss	AI vs pathologist consensus	685	0.69 (0.65–0.73)	0.82 (0.79–0.85)
	AI vs pathologist 1 (AE)	601*	0.66 (0.62–0.71)	0.79 (0.76–0.83)
	AI vs pathologist 2 (AE)	601*	0.66 (0.62–0.71)	0.79 (0.75–0.82)
	Pathologist 1 vs 2	601*	0.84 (0.81–0.88)	0.91 (0.88–0.93)
Predicted values† in total study population				
MMR status (retained vs lost)	AI vs pathologist consensus	685 analysed 1,331 predicted†	0.85 (0.82–0.88)	0.96 (0.95–0.97)
Individual MMR protein loss	AI vs pathologist consensus	601 analysed 1,331 predicted†	0.75 (0.71–0.79)	0.94 (0.93–0.95)

Individual pathologist review of MMR status and individual MMR protein expression was performed blinded to results of AI-based analysis and the interpretation of the other pathologist. All cases with discordance between AI and one or both pathologists, as well as all cases where individual pathologists were discordant were reviewed at a discrepancy meeting, with final status resolved by discussion. *excludes subset of cases with consensus pathology review but not blinded individual pathological review. †Predicted values are calculated based on the assumption of equivalent concordance between AI and pathologist review in all 1,529 cases with ≥20% epithelial cells positive for all MMR proteins as that obtained by comparison of the randomly-selected subset of 198 cases (100% agreement).

Table S3. Classification of MMR protein loss by AI and consensus pathology review by type of MMR loss. Related to Figure 3.

AI classification		Consensus pathologist classification		PPV for MMR status	PPV for protein loss
**Group**	**Number**	**Group**	**Number**		
MLH1 & PMS2 or MSH2 & MSH6 loss	147	MLH1/PMS2 or MSH2/MSH6	140	0.966	0.952*
		single protein	2		
		Other loss	0		
		retained	2		
		fail	3		
Single MMR protein loss	89	MLH1/PMS2 or MSH2/MSH6	35	0.618	0.213†
		single protein	19		
		other loss	1		
		retained	22		
		fail	12		
Other MMR loss	26	MLH1/PMS2 or MSH2/MSH6	11	0.577	0.115‡
		single protein	1		
		other loss	3		
		retained	1		
		fail	10		
MMRp	423	MLH1/PMS2 or MSH2/MSH6	14	0.955	0.955§
		single protein	3		
		Other loss	0		
		retained	404		
		fail	2		

* denotes PPV for combined MLH1 &PMS2 loss or combined MSH2 & MSH6 loss; † denotes PPV for single MMR protein loss; ‡ denotes PPV for other patterns of MMR protein loss; § denotes PPV for retained MMR expression

Table S4. Reasons for discordance between AI and consensus pathologist classification. Related to Figure 3.

Reason for discordance	MMR status discordant		MMR status concordant but protein loss discordant	
	N	%	N	%
Immunostaining failed	27	39.1	NA	NA
Immunostaining weak/heterogenous	34	49.3	35	70.0
Background/cytoplasmic immunostaining	1	1.4	2	4.0
Technical: tissue folded	1	1.4	0	0.0
Technical: shadow/out of focus	2	2.9	4	8.0
Atypical epithelial morphology	2	2.9	2	4.0
Subclonal alteration	1	1.4	5	10.0
No reason identified	1	1.4	2	4.0

Table S5. Reasons for discordance between individual pathologist classification. Related to Figure 3.

Reason for discordance	MMR status discordant		MMR status concordant but protein loss discordant	
	N	%	N	%
Immunostaining failed	18	47.4	0	NA
Immunostaining weak/heterogenous	11	28.9	8	50
Background/cytoplasmic immunostaining	0	0	1	6.3
Technical: tissue folded	1	2.6	0	0
Technical: shadow/out of focus	3	7.9	0	0
Technical: other	2	5.3	1	6.3
Atypical epithelial morphology	1	2.6	0	0
Subclonal alteration	2	5.3	0	0
No reason identified	0	0	6	37.5

Table S6. Clinicopathological characteristics of SCOT trial by MMR status. Related to Figure 5.

	MMRp		MMRd		p-value[2]
Total	1,759		229		
Age					
Median IQR					<0.001
<70	1,302	74	138	60	
>/70	457	26	91	40	
Gender					<0.001
Female	669	38	133	58	
Male	1,090	62	96	42	
Performance status					
0-1	1,759	100	229	100	1.0
≥2	0	0	0	0	
pT stage					<0.001
1-2	137	7.8	3	1.3	
3	1,042	59	131	57	
4	541	31	95	41	
Unknown	39	2.2	0	0	
N stage					<0.001
0	315	18	76	33	
1	946	54	109	48	
2	457	26	44	19	
NA	39	2.2	0	0	
AJCC disease stage					<0.001
2	314	18	76	33	
3	1,402	80	152	66	
Unknown	42	2.4	0	0	
Stage III Risk Status					<0.001
High	690	39	76	33	
Low	712	40	76	33	
Not applicable (stage II)	357	20	77	33	
Tumour Location					<0.001
Left	1,144	65	36	16	
Right	586	33	193	84	
Unknown	29	1.6	0	0	
Treatment Regimen					0.5
CAPOX	1,241	71	157	69	
FOLFOX	518	29	72	31	
Treatment duration					0.2
12 weeks	876	50	124	54	
24 weeks	883	50	105	46	

pT –pathological tumour (T) stage; MMR – DNA mismatch repair; MMRp – mismatch repair proficient; MMRd – mismatch repair deficient;
*determined by unpaired Student's t-test.
†determined by Fisher exact test (in cases which marker status was determined).

Table S7. Univariable and multivariable analysis of recurrence-free interval in SCOT trial cohort according to clinicopathologic factors and MMR status. Related to Figure 5.

	Univariate					Multivariate				
**Characteristic**	**Cases**	**Events**	**HR[1]**	**95% CI[1]**	***P***	**Cases**	**Events**	**HR[1]**	**95% CI[1]**	***P***
Age	1,959	464				1,942	461			
<70			1.00	—				1.00	—	
>/70			1.03	0.84, 1.26	0.78			0.97	0.79, 1.19	0.8
Gender	1,959	464				1,942	461			
F			1.00	—				1.00	—	
M			0.96	0.79, 1.15	0.63			0.97	0.80, 1.17	0.7
Tumour Stage	1,945	463				1,942	461			
1-2			1.00	—				1.00	—	
3			2.75	1.50, 5.04	0.001			2.76	1.50, 5.07	0.001
4			5.29	2.88, 9.69	<0.001			5.27	2.85, 9.74	<0.001
Nodal Stage	1,943	461				1,942	461			
0			1.00	—				1.00	—	
1			1.41	1.05, 1.87	0.020			1.74	1.29, 2.33	<0.001
2			2.83	2.11, 3.79	<0.001			3.06	2.28, 4.12	<0.001
Sidedness	1,959	464				1,942	461			
Left			1.00	—				1.00	—	
Right			1.41	1.17, 1.69	<0.001			1.30	1.07, 1.58	0.008
Regimen	1,959	464				1,942	461			
Capox			1.00	—				1.00	—	
Folfox			1.11	0.92, 1.35	0.28			1.01	0.83, 1.23	>0.9
Duration	1,959	464				1,942	461			
12 weeks			1.00	—				1.00	—	
24 weeks			0.91	0.75, 1.09	0.28			0.92	0.76, 1.10	0.4
MMR status	1,959	464				1,942	461			
MMRp			1.00	—				1.00	—	
MMRd			0.69	0.50, 0.96	0.029			0.62	0.44, 0.88	0.007

[1]HR = Hazard Ratio, CI = Confidence Interval

Table S8. Biomarker analyses performed and reported in this study. Related to STAR methods.

Analysis	Population	Methods	Reported
RFI by MMR status	Stage II/III CRCs from SCOT trial	Log-rank test, univariable and multivariable adjusted HR	Main text, Figure 5, Table S8
RFI by MMR status	Defined subgroups in SCOT trial	Univariable and multivariable adjusted HR	Figure 5<br>Table S8

RFI – recurrence-free interval; OS – overall survival; HR – hazard ratio. *Full multivariable model included age, gender, location,

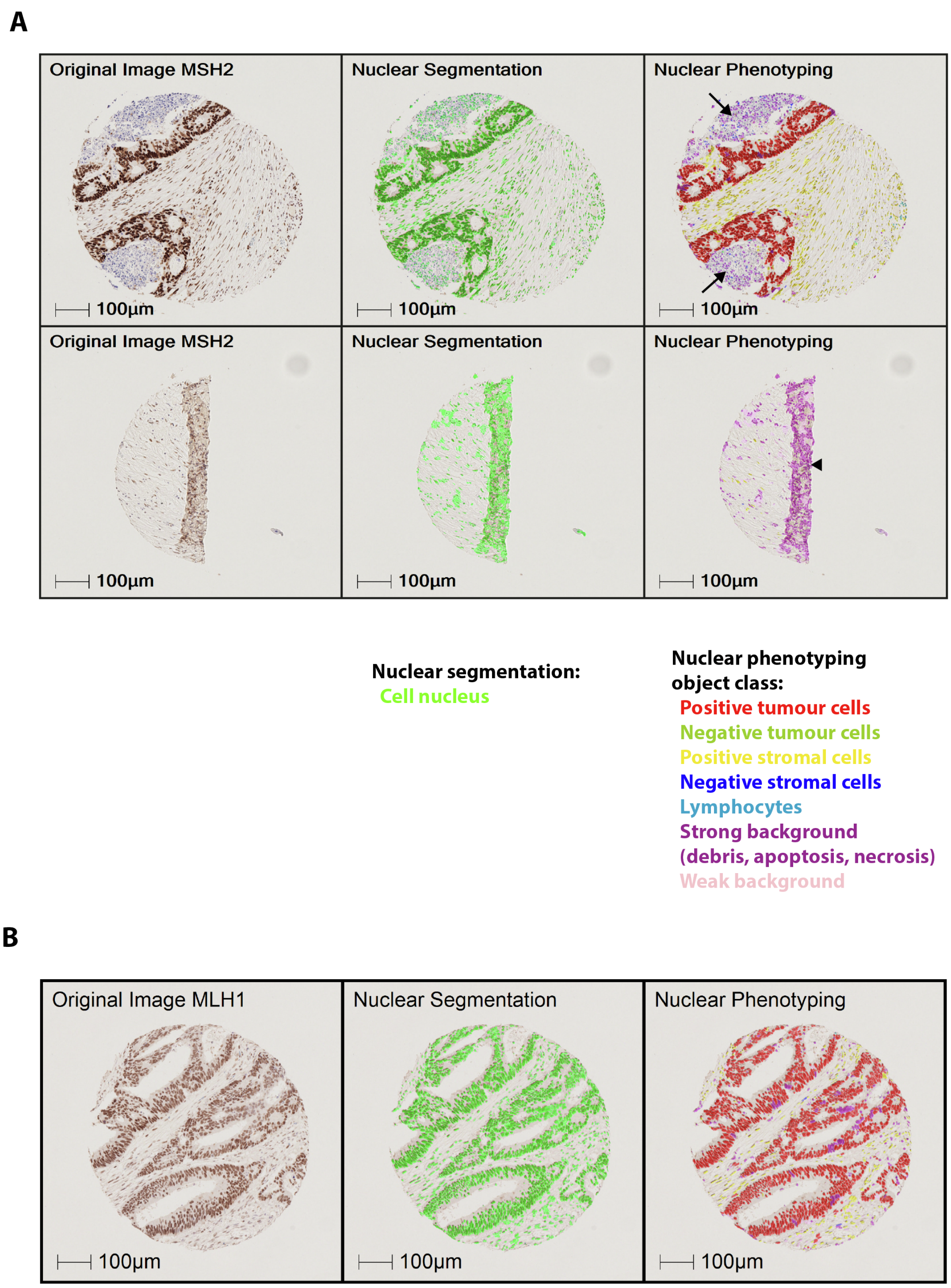

Figure S1. Nuclear segmentation by object class. Related to STAR methods.
(A) Illustrative images of AI-based classification of cell nuclei and other objects on tumor sections following IHC for MMR proteins. Panels show the original IHC stained images (left), nuclear segmentation mark-ups (centre) and nuclear phenotyping by object classification (right). Upper panels show the original image (left), nuclear segmentation (center) and classification results (right) of a representative tissue microarray core with tumor cells, stromal cells and intraglandular debris (black arrows) correctly classified . Lower panels show an exemplary core with a folding artefact (black arrow), with the nuclei in the affected area and adjacent out-of-focus tissue regions correctly classified as strong background (uninformative for analysis) by nuclear phenotyping,. Note that partial transparency in nuclear segmentation masks permits visualisation of DAB staining in MSH2 positive cells.

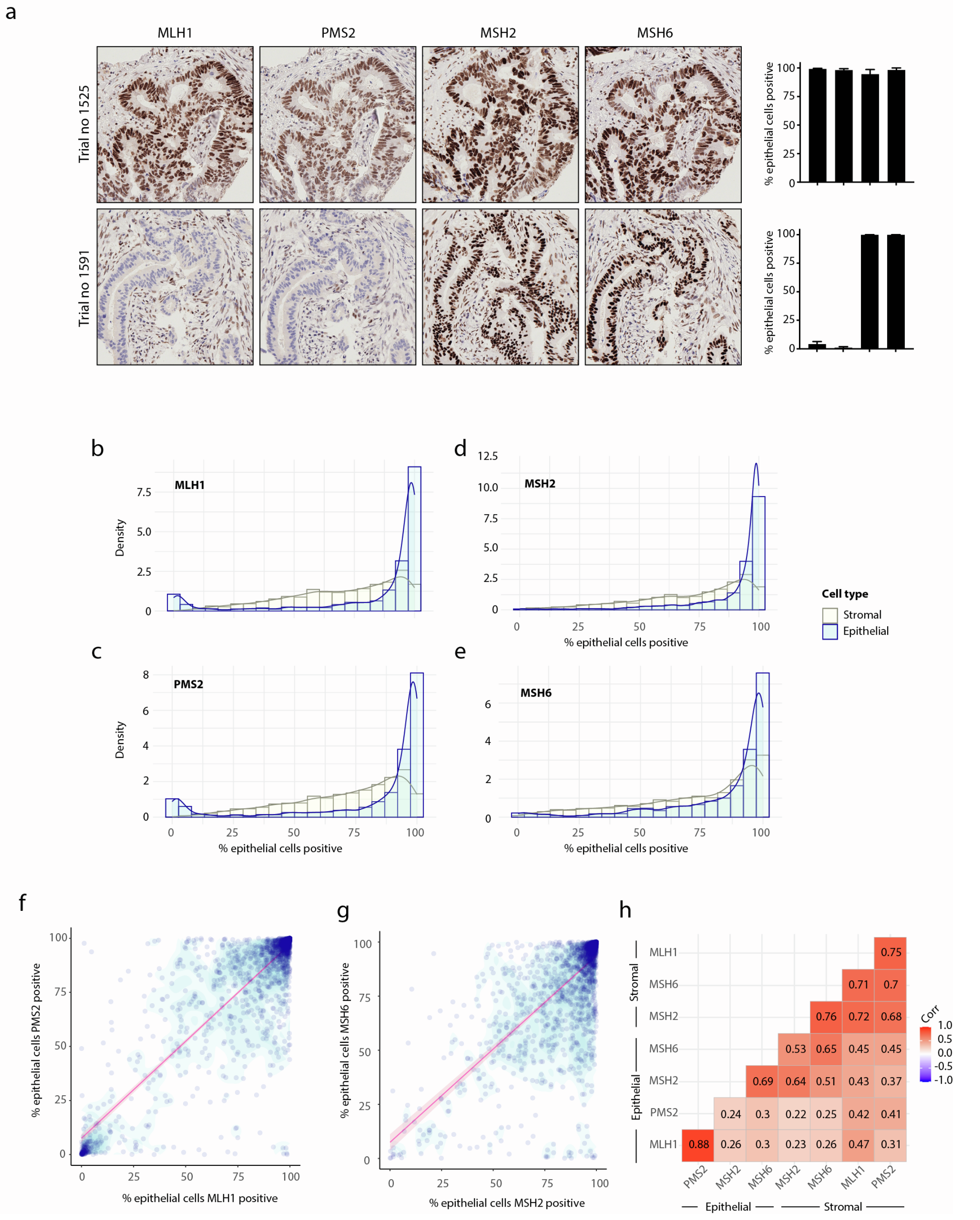

Figure S2. AIMMeR single cell analysis of MMR proteins identifies loss of epithelial expression and expected correlations. Related to Figure 3.

(a) Representative images of immunohistochemistry (IHC) for DNA mismatch repair (MMR) proteins MLH1, PMS2, MSH2 and MSH6 in cases with expression of all proteins (upper row), and loss of MLH1 and PMS2 with retained expression of MSH2 and MSH6 (lower row). Barplots to the right show the percentage of epithelial cells positive for each MMR protein as determined by AI (error bars indicate standard deviation between cores). (b-d) Frequency histograms with overlaid kernel density plots showing proportion of cases by percentage of epithelial and stromal cells positive for MMR proteins MLH1 (b), PMS2 (c), MSH2 (d) and MSH6 (e). (f, g) Scatterplots showing relationship between percentage of epithelial cells expressing dimerization partners MLH1 and PMS2 (f) and MSH2 and MSH6 (g). Regression line represents Spearman rho with 95% confidence intervals. (h) Matrix showing correlation between epithelial and stromal cell positivity for all four MMR proteins (P<2.2e-16 all cases). For consistency, plots in (b-h) show results from analysis of 1,988 cases and exclude 27 cases classed as failed on pathologist review; plots from analysis of original 2,015 cases were essentially identical.

.

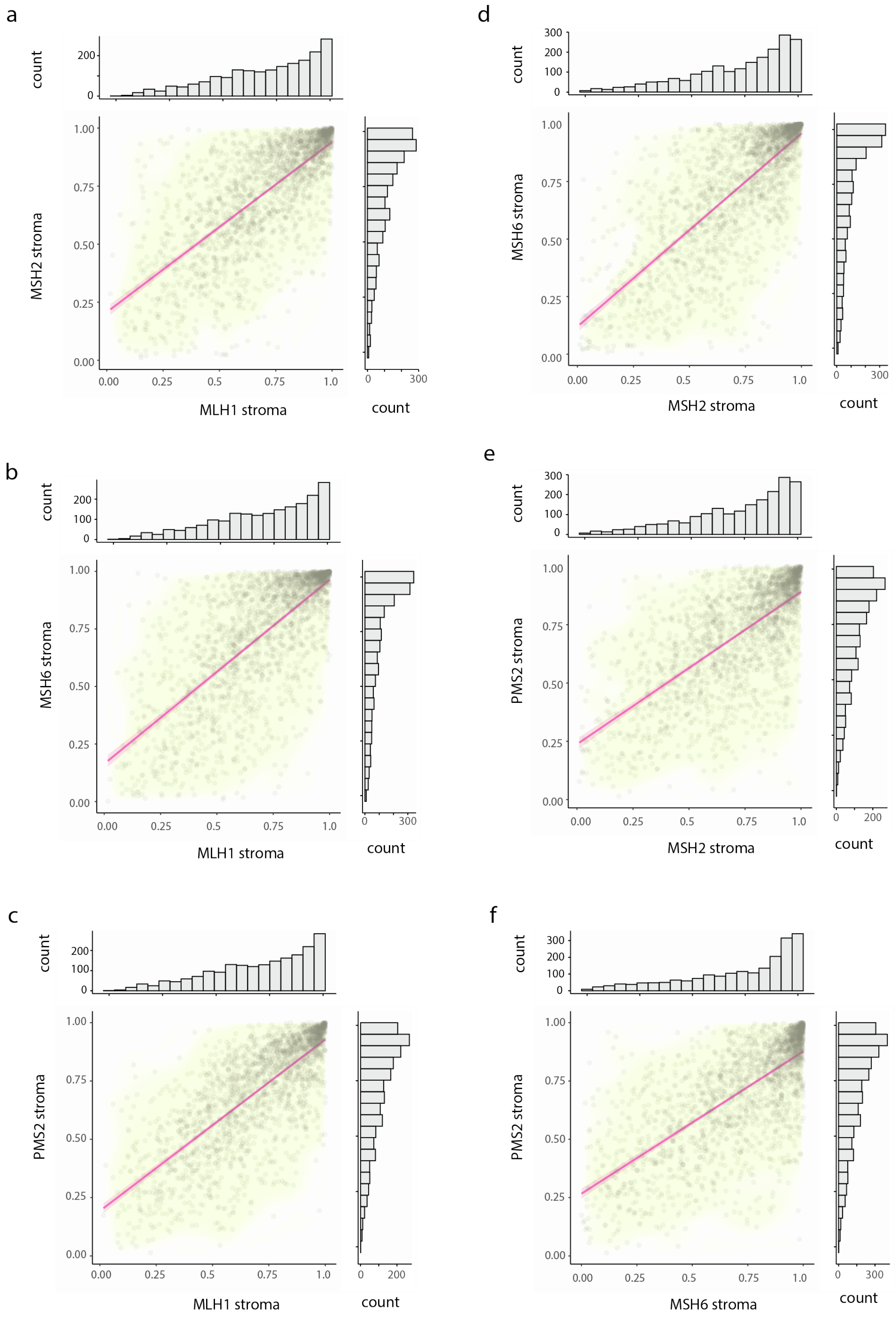

Figure S3. Scatterplots showing correlation between stromal MMR protein expression across cases. Related to Figure 3.

Scatterplots with marginal histograms showing correlation between the proportion of stromal cells positive for individual MMR proteins for: (a) MLH1 and MSH2, (b) MLH1 and MSH6, (c) MLH1 and PMS2; (d) MSH2 and MSH6; (e) MSH2 and PMS2 and: (f) MSH6 and PMS2.

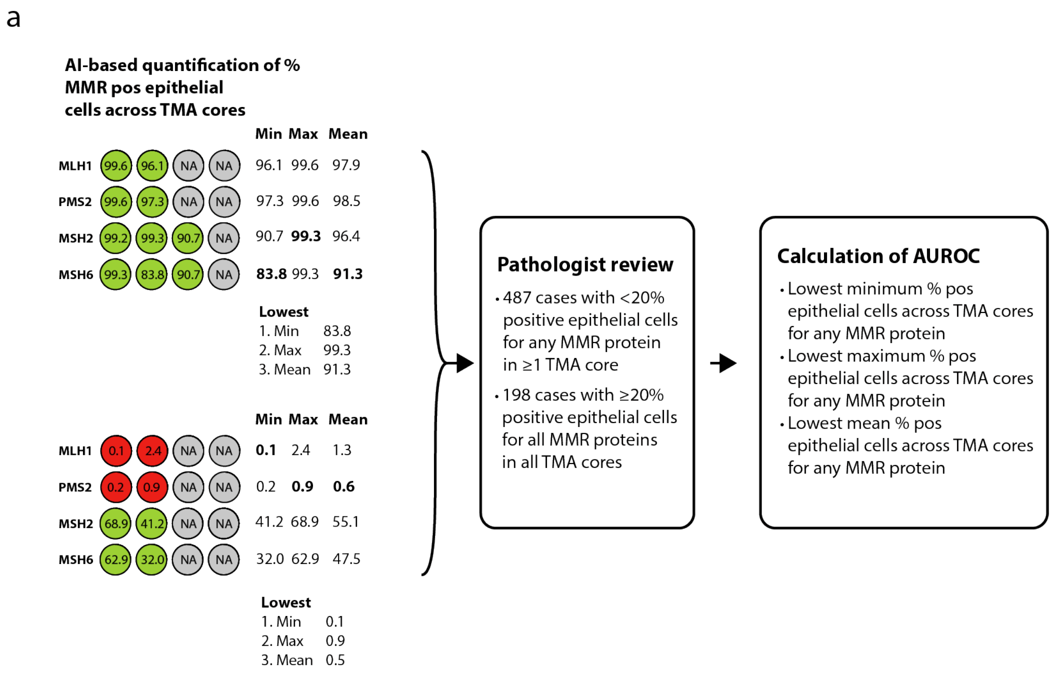

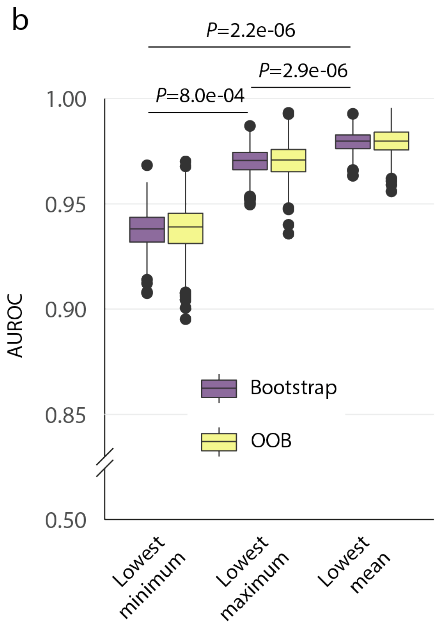

Figure S4. Calculation of AUROC for alternative methods for classification of MMR loss. Related to Figure 3.

(a) Schematic showing study workflow and identification of cases for establishing consensus pathologist ground truth for evaluation of AIMMeR performance. (b) AUROC calculated against consensus pathologist ground truth using alternative metrics based on minimum, maximum and mean percentage of cells positive for individual MMR proteins. Boxplots show median, 25th and 75th percentiles ± 1.5 x interquartile range and outlying points obtained from bootstrap with 1000 resamples and corresponding out of bag (OOB estimates). *P* values were obtained by Mann-Whitney U test.

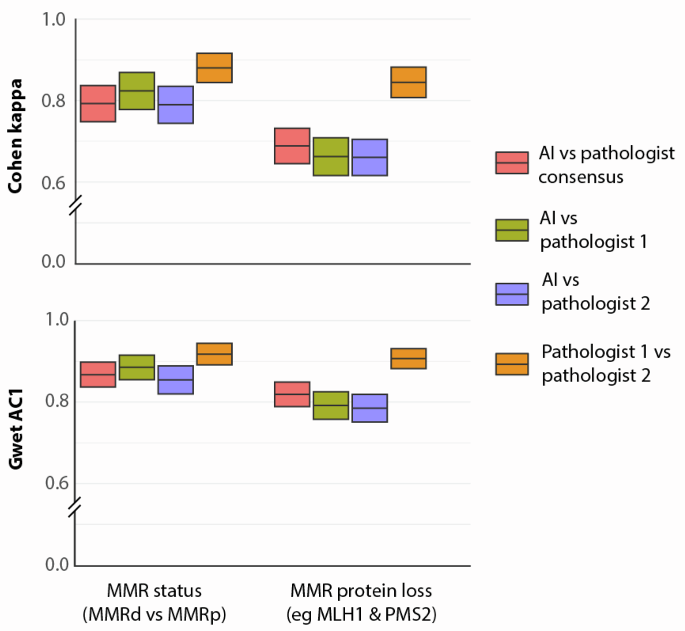

Figure S5. Agreement in classification of MMRd and individual protein loss between AIMMeR, individual and consensus pathologist calls. Related to Figure 3.

Inter-rater reliability measures of agreement between AIMMeR classification of tumour MMR status (left) and combination of MMR protein loss (right) vs individual and consensus pathologist classification. Measures of between-pathologist agreement are shown for comparison. AIMMeR classification uses AIMMeR^{MIN} threshold with maximal Youden index to define MMRd and individual protein loss.

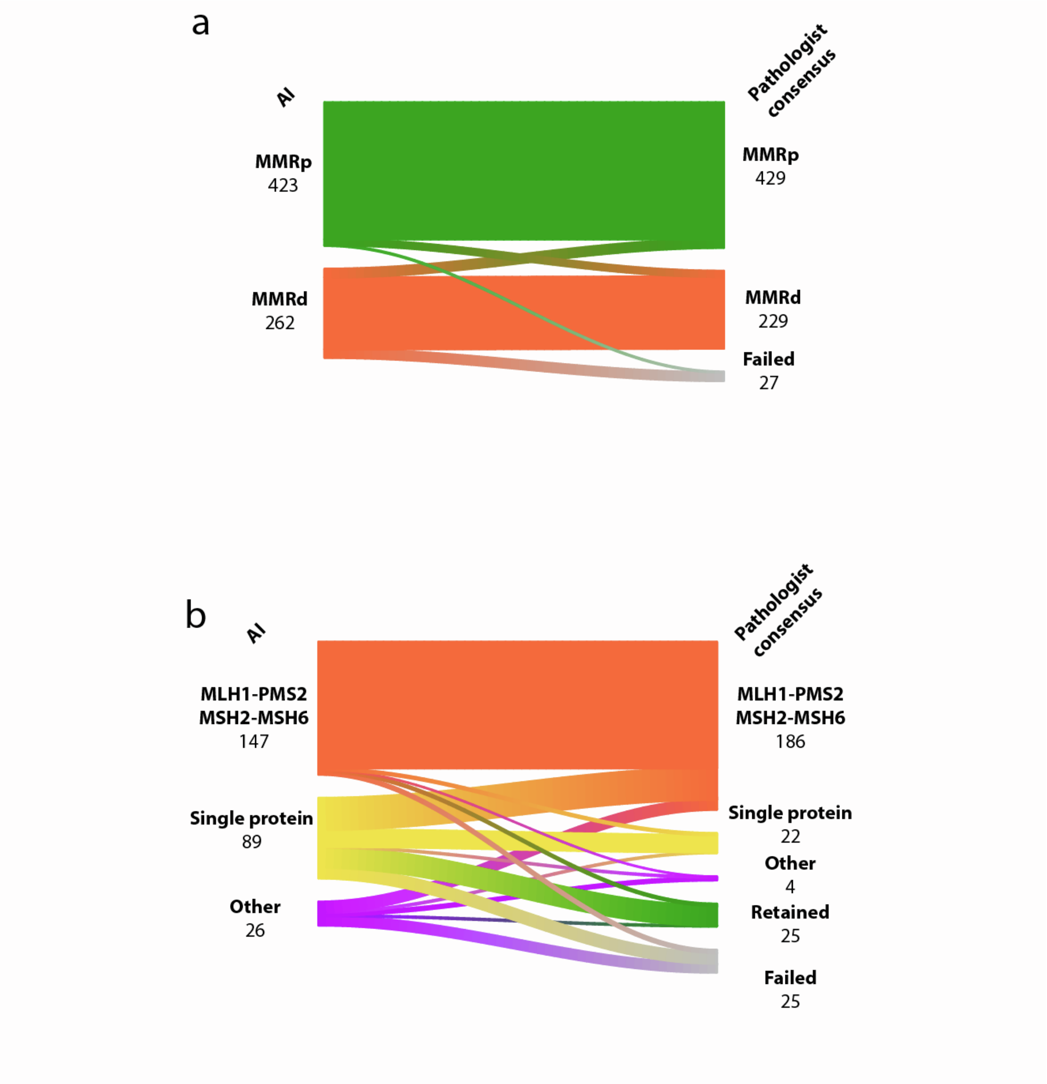

Figure S6. Sankey plots showing relationship between AIMMeR classification of MMR status and individual MMR protein loss. Related to Figure 3.
(a) Plot showing relationship between AIMMeR classification and consensus pathologist review for (a) MMR status and (b) pattern of protein loss. Category of "other" includes MLH1-PMS2 or MSH2-MSH6 loss plus other MMR proteins as well as alternative combinations of loss. AIMMeR calls used AIMMeRMIN threshold with maximal Youden index.

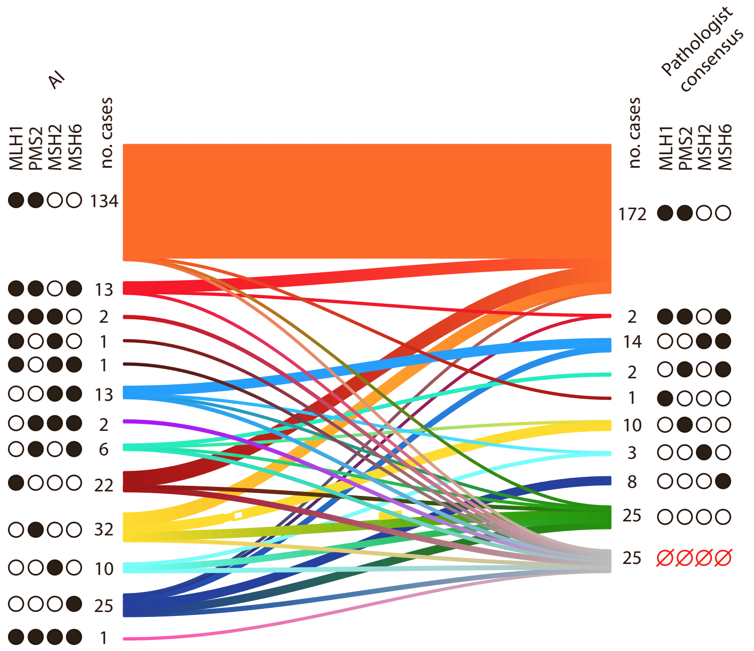

Figure S7. Relationship between AI and consensus pathologist calls for combinations of MMR protein loss. Related to Figure 3.
Sankey plot showing flow between initial AI classification of MMR protein loss using cutpoint of 10.7% positive epithelial cells and final consensus pathologist calls. Proteins lost are indicated by closed circles. Open red circles indicate failed cases.

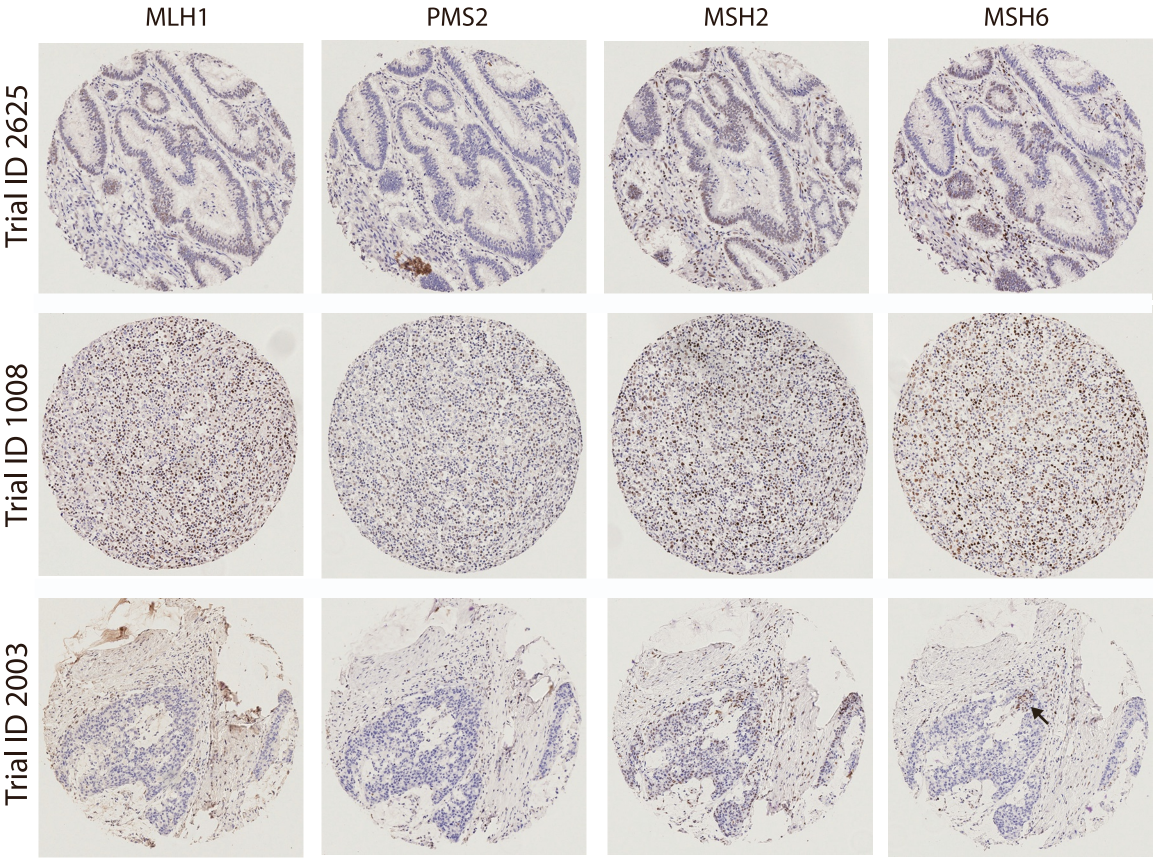

Figure S8. Cases discordant between AI-based and pathologist classification. Related to Figure 3. Representative cases illustrative of causes of AI-pathologist discordance are shown. Upper panel shows failed immunostaining in tumour from participant ID 2625. Immunostain for PMS2 was classified by AI as negative (no positive tumour cells). On pathologist review, this was reclassified to failed staining in view of absence of internal positive controls, and poor quality immunostaining for other MMR proteins. Middle panels show a case misclassified by AI as MMRp as a result of atypical tumour epithelial morphology, in setting of intense lymphocytic infiltrate. On pathologist review, this was reclassified to MMRd with loss of MLH1 and PMS2. Lower panels show a case classified as MMRd by both AI and pathologist review, but discordant for protein loss. AI-based analysis classified this as lacking expression of MLH1, PSM2 and MSH6; the latter owing to MSH6 expression in 2.6% of epithelial cells. Pathologist review identified a small area of retained MSH6 expression (black arrow) in background of loss, leading to reclassification of case as MLH1 and PMS2 deficient with subclonal MSH6 loss.

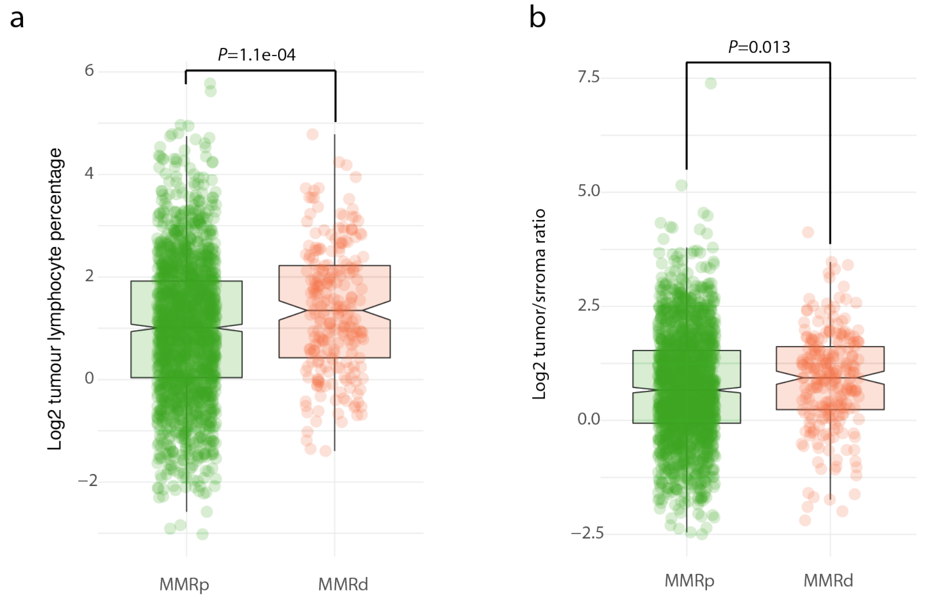

Figure S9. Tumor lymphocytic infiltrate and tumor/stroma ratio by MMR status. Related to Figure 5.
(a) Lymphocytes as percentage of all cells within tumor (determined by single-cell AI-based analysis) according to MMR status. (b) Tumor (malignant epithelial) cell/stroma cell ratio (determined by single-cell AI-based analysis) according MMR status.
