# Supplementary material for: Single-cell AI-based detection and prognostic and predictive value of DNA mismatch repair deficiency in colorectal cancer
Source: Cell Rep Med. 2024 Sep 17;5(9):101727. doi: 10.1016/j.xcrm.2024.101727 (PMC11525017; doi:10.1016/j.xcrm.2024.101727)
Supplement: Document S2. TransSCOT Trial Management Group members and affiliations [file mmc2.pdf]

Secondary author affiliations

Bengt Glimelius[1], Ismail Gogenur[2], Emma Jaeger[3], Hannah Morgan[4], Clare Orange[5], Claire Palles[6], Campbell Roxburgh[7]

[1]Uppsala University, Uppsala, Sweden
[2]Centre for Surgical Science, Zealand University Hospital, Denmark
[3]Department of Oncology, University of Oxford, UK
[4]Glasgow Tissue Research Facility, University of Glasgow, Queen Elizabeth University Hospital, Glasgow, UK
[5]NHS Greater Glasgow and Clyde Biorepository, Glasgow, UK
[6]University of Birmingham, Birmingham, UK
[7]School of Cancer Sciences, University of Glasgow, Glasgow, UK

TransSCOT consortium affiliations

David Church[1], Enric Domingo[2], Joanne Edwards[3], Bengt Glimelius[4], Ismail Gogenur[5], Andrea Harkin[6], Jen Hay[7], Timothy Iveson[8], Emma Jaeger[2], Caroline Kelly[6], Rachel Kerr[2], Noori Maka[7], Hannah Morgan[7], Karin Oien[7], Clare Orange[9], Claire Palles[10], Campbell Roxburgh[3], Owen Sansom[11], Mark Saunders[12], Ian Tomlinson[2].

[1]Cancer Genomics and Immunology Group, The Centre for Human Genetics, University of Oxford UK
[2]Department of Oncology, University of Oxford, UK
[3]School of Cancer Sciences, University of Glasgow, Glasgow, UK
[4]Uppsala University, Uppsala, Sweden
[5]Centre for Surgical Science, Zealand University Hospital, Denmark
[6]CRUK Glasgow Clinical Trials Unit, University of Glasgow, Glasgow, UK
[7]Glasgow Tissue Research Facility, University of Glasgow, Queen Elizabeth University Hospital, Glasgow, UK
[8]University of Southampton, Southampton, UK
[9]NHS Greater Glasgow and Clyde Biorepository, Glasgow, UK
[10]University of Birmingham, Birmingham, UK
[11]CRUK Beatson Institute of Cancer Research, Garscube Estate, Glasgow, UK
[12]The Christie NHS Foundation Trust, Manchester, UK
