# Supplementary material for: Single-cell AI-based detection and prognostic and predictive value of DNA mismatch repair deficiency in colorectal cancer
Source: Cell Rep Med. 2024 Sep 17;5(9):101727. doi: 10.1016/j.xcrm.2024.101727 (PMC11525017; doi:10.1016/j.xcrm.2024.101727)
Supplement: Document S3. Article plus supplemental information [file mmc3.pdf]

Cell Reports
Medicine

Article

Single-cell AI-based detection and prognostic and predictive value of DNA mismatch repair deficiency in colorectal cancer

Graphical abstract

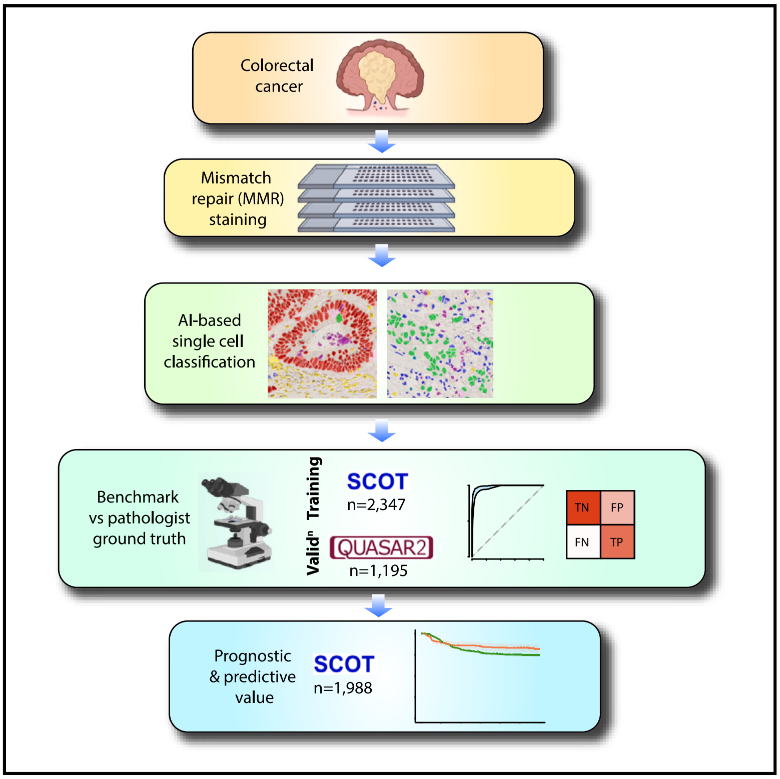

Authors

Marta Nowak, Faiz Jabbar, Ann-Katrin Rodewald, ..., TransSCOT group, Viktor H. Koelzer, David N. Church

Correspondence

dchurch@well.ox.ac.uk

In brief

Nowak et al. report the development and testing of an AI-based method (AIMMeR) for detection of DNA mismatch repair loss in colorectal cancer at a single-cell level. AIMMeR shows an AUROC of 0.98 in two independent cohorts and holds promise for clinical implementation.

Highlights

- AIMMeR is an AI-based method for classification of MMR status at a single-cell level
- AIMMeR has an AUROC of 0.98 vs. pathologist ground truth in two independent CRC trials
- AIMMeR holds potential to automate diagnostic workflows and refine stratification
- MMR deficiency is prognostic in oxaliplatin-treated patients

Nowak et al., 2024, Cell Reports Medicine *5*, 101727
September 17, 2024 © 2024 The Author(s). Published by Elsevier Inc.
https://doi.org/10.1016/j.xcrm.2024.101727

CellPress

Cell Reports Medicine

CellPress OPEN ACCESS

Article

Single-cell AI-based detection and prognostic and predictive value of DNA mismatch repair deficiency in colorectal cancer

Marta Nowak,[1] Faiz Jabbar,[2] Ann-Katrin Rodewald,[1] Luciana Gneo,[2] Tijana Tomasevic,[2] Andrea Harkin,[3] Tim Iveson,[4] Mark Saunders,[5] Rachel Kerr,[6] Karin Oein,[7] Noori Maka,[7] Jennifer Hay,[7] Joanne Edwards,[8] Ian Tomlinson,[6] Owen Sansom,[9] Caroline Kelly,[3] Francesco Pezzella,[10] David Kerr,[11] Alistair Easton,[6] Enric Domingo,[6] TransSCOT group, Viktor H. Koelzer,[1,6,12,13,15] and David N. Church[2,14,15,16,*]

[1]Department of Pathology and Molecular Pathology, Zurich, Zurich, Switzerland
[2]Cancer Genomics and Immunology Group, The Wellcome Centre for Human Genetics, University of Oxford, Roosevelt Drive, Oxford OX3 7BN, UK
[3]CRUK Glasgow Clinical Trials Unit, University of Glasgow, Glasgow, UK
[4]University of Southampton, Southampton, UK
[5]The Christie NHS Foundation Trust, Manchester, UK
[6]Department of Oncology, University of Oxford, Oxford, UK
[7]Glasgow Tissue Research Facility, University of Glasgow, Queen Elizabeth University Hospital, Glasgow, UK
[8]School of Cancer Sciences, University of Glasgow, Glasgow, UK
[9]CRUK Beatson Institute of Cancer Research, Garscube Estate, Glasgow, UK
[10]Radcliffe Department of Medicine, University of Oxford, Oxford, UK
[11]Nuffield Department of Clinical and Laboratory Sciences, University of Oxford, Oxford, UK
[12]Nuffield Department of Medicine, University of Oxford, Oxford, UK
[13]Institute of Medical Genetics and Pathology, University Hospital Basel, Basel, Switzerland
[14]Oxford NIHR Comprehensive Biomedical Research Centre, Oxford University Hospitals NHS Foundation Trust, Oxford, UK
[15]These authors contributed equally
[16]Lead contact
*Correspondence: dchurch@well.ox.ac.uk
https://doi.org/10.1016/j.xcrm.2024.101727

SUMMARY

Testing for DNA mismatch repair deficiency (MMRd) is recommended for all colorectal cancers (CRCs). Automating this would enable precision medicine, particularly if providing information on etiology not captured by deep learning (DL) methods. We present AIMMeR, an AI-based method for determination of mismatch repair (MMR) protein expression at a single-cell level in routine pathology samples. AIMMeR shows an area under the receiver-operator curve (AUROC) of 0.98, and specificity of ≥75% at 98% sensitivity against pathologist ground truth in stage II/III in two trial cohorts, with positive predictive value of ≥98% for the commonest pattern of somatic MMRd. Lower agreement with microsatellite instability (MSI) testing (AUROC 0.86) reflects discordance between MMR and MSI PCR rather than AIMMeR misclassification. Analysis of the SCOT trial confirms MMRd prognostic value in oxaliplatin-treated patients; while MMRd does not predict differential benefit of chemotherapy duration, it correlates with difference in relapse by regimen ($P_{\text{Interaction}}$ = 0.04). AIMMeR may help reduce pathologist workload and streamline diagnostics in CRC.

INTRODUCTION

Colorectal cancer (CRC) is the third most common tumor globally, and a substantial cause of morbidity and mortality.[1] 10%–15% of CRCs display genomic instability due to DNA mismatch repair deficiency (MMRd), caused by germline mutation of mismatch repair (MMR) genes *MLH1*, *MSH2*, *MSH6*, or *PMS2* (Lynch syndrome),[2] biallelic somatic mutation of MMR genes,[3,4] or, more commonly, somatic silencing of *MLH1* by promoter methylation.[5] MMR deficiency (MMRd) causes failure to repair errors accumulated during DNA replication—particularly those at error-prone DNA microsatellites—resulting in elevated tumor mutational burden and microsatellite instability (MSI). MMRd CRC displays characteristic clinical and pathological features, including right-sided colonic location, female preponderance, prominent lymphocytic infiltrate, and good prognosis in early-stage disease.[6–9] They are also highly sensitive to immune checkpoint blockade, resulting in prolonged disease control in the metastatic setting,[10,11] and unprecedented pathological responses in localized disease that have raised the possibility of organ-sparing therapy.[12,13] In view of these important correlates, reflex immunohistochemistry (IHC) for MMRd or MSI PCR is recommended for

This is an open access article under the CC BY license (http://creativecommons.org/licenses/by/4.0/).

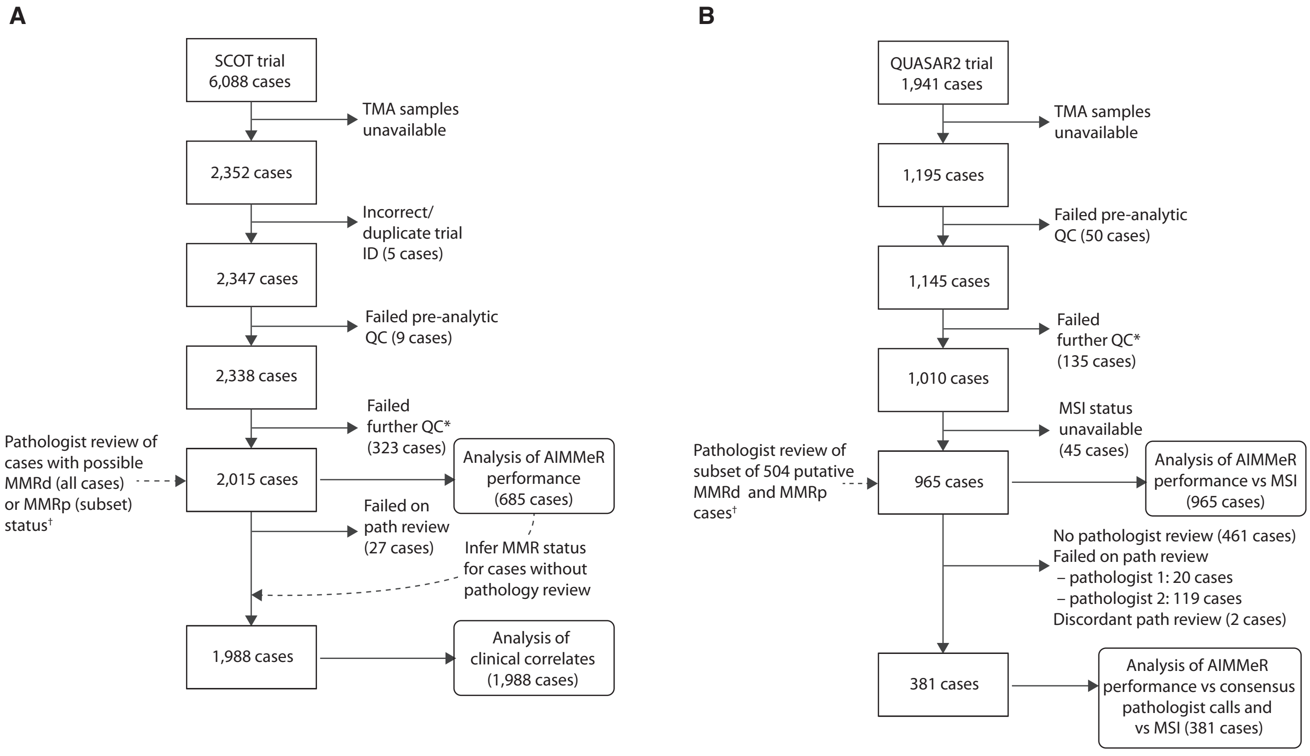

Figure 1. Flow (CONSORT) diagram of cases included in this study

CONSORT diagrams for (A) SCOT and (B) QUASAR2 trial cases. *Additional QC included exclusion of tissue microarray (TMA) cores with <100 epithelial cells per core, cores with negative staining (<20 positive cells) for all four MMR proteins in both epithelium and stroma, and cases uninformative for all four MMR proteins. †Details of cases subject to pathologist review are provided in the STAR Methods.

all incident CRCs by international guidelines.[14–16] In most centers, MMR IHC is preferred, with slides or images reviewed by specialized gastrointestinal (GI) pathologists and MMRd identified by loss of MMR protein expression in tumor epithelium. This requires substantial pathologist time. Consequently, efforts have focused on the development of automated methods to identify MMRd, leveraging advances in artificial intelligence (AI) and deep learning (DL) for image analysis.[17–29] In a proof-of-principle study, Kather and colleagues developed an AI-based DL method to identify MMRd in gastrointestinal cancers from hematoxylin and eosin (H&E) stained slides.[17] Subsequent refinement in larger CRC series has improved area under the receiver-operator curve (AUROC) to as high as 0.97.[18,23,27,29] While impressive, when used at a fixed 95% sensitivity, the limited specificity of these methods (41% in the largest study to date[29]) means that MMR testing and pathologist review are still required in between 23.2% and 87.4% cases.[23,27,29] Furthermore, such methods provide no information about the likely etiology of MMRd, detail regarding which is helpful in stratifying cases for germline testing.[30,31]

One possible alternative to H&E slides would be to use IHC-stained images of tumor tissue as the substrate for automated image analysis. Multiple studies, including from our group, have published methods for quantification of IHC-labeled cells in whole tumors[32] or defined intratumoral regions such as the tumor invasive margin [33] and intraepithelial or intrastromal compartments.[34] While defining MMR protein expression in cells within the tumor intraepithelial compartment is superficially attractive for detection of MMRd, this approach is undermined by the detection of intraepithelial lymphocytes, which retain MMR protein expression and are enriched in MMRd tumors.[32,35,36] This shortcoming may be circumvented by the classification of MMR protein expression in individual cells; however, methods to do this have previously been lacking. We sought to address this by developing AIMMeR—AI-based analysis of MMR status at the single-cell level. We benchmarked AIMMeR performance against the gold standard of pathologist review in more than 1,000 cases across training and validation cohorts from the Short Course Oncology Treatment (SCOT)[37] and Quick and Simple and Reliable (QUASAR2)[38] clinical trials and used determined MMRd prognostic and predictive value in the practice-defining SCOT study.

RESULTS

Development of AIMMeR: An AI-based methodology for single-cell analysis of DNA MMR loss

To develop AIMMeR (AI-based method to detect MMRd), we first performed immunostaining for MMR proteins MLH1, MSH2, MSH6, and PMS2 on tumor tissue microarrays (TMAs) from the SCOT trial, which compared 6 vs. 3 months of adjuvant oxaliplatin-based chemotherapy in stage III and high-risk stage

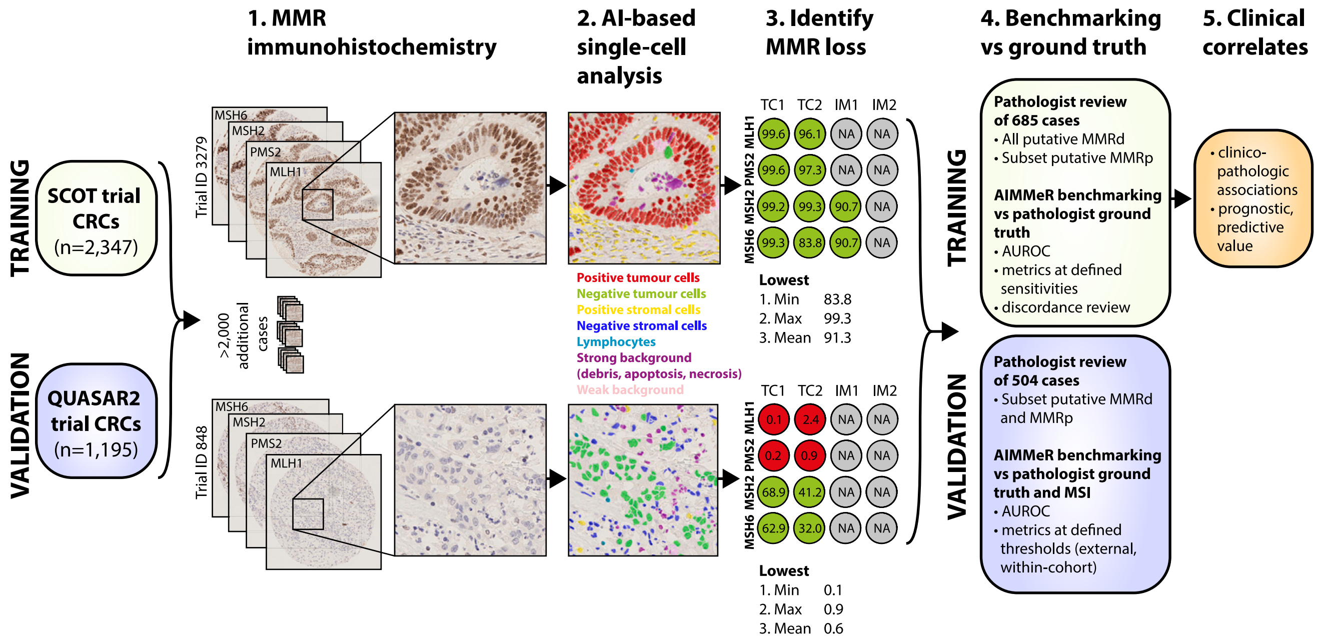

Figure 2. Schematic of study methodology

Immunohistochemistry (IHC) for DNA mismatch repair (MMR) proteins MLH1, PMS2, MSH2, and MSH6 was performed on tissue microarray (TMA) cores from 2,352 SCOT and 1,195 QUASAR2 tumors. Scanned IHC images were classified according to type and MMR staining by AIMMeR (see STAR Methods and Results). Following QC, the percentage of epithelial cells positive for expression of individual MMR proteins was calculated for each core, and summary metrics were calculated for each case (Step 3). AIMMeR performance was then benchmarked against a ground-truth set defined by pathologist review of 685 SCOT tumors and 504 QUASAR2 tumors and against MSI status in QUASAR2 cases. Further details are provided in the STAR Methods and the main text. Min, minimum; Max, maximum; AUROC, area under the receiver-operator curve; IRR, inter-rater reliability.

II CRC across >100 recruiting sites[37] (study CONSORT diagram provided as Figure 1). As misclassification of MMR protein-expressing non-epithelial cells (e.g., lymphocytes) in the intraepithelial compartment could confound assignment of MMR status, we developed a method to classify cells by their nuclear morphology (epithelial, stromal, lymphocyte) with accuracy of 0.92 against pathologist ground truth (STAR Methods, Figure S1). This method identified other relevant features, including non-nuclear objects such as apoptotic bodies, and artifacts including tissue folds and background staining (Figure S1). We combined the nuclear classifier with automated identification of 3-3′diaminobenzidine (DAB) signal[34] and used the combined method—AIMMeR—to determine expression of individual MMR proteins in single cells, and thus the percentage of MMR-expressing epithelial and stromal cells in TMA cores and cases (Figure 2). After removing cases with duplicate or non-matching trial ID, pre-analytic fails, and those failing rigorous quality control (QC) (STAR Methods), 2,015 SCOT tumors and 38,113,216 single cells were informative for the initial evaluation of AIMMeR performance.

AIMMeR quantification of epithelial cell MMR proteins revealed homogeneous expression ($\geq$90% cells positive) of all four proteins in two-thirds of tumors, and low or absent expression (<10% cells positive) in $\sim$10% cases, most commonly for MLH1 and PMS2 (Figure S2). Strong positive correlations in epithelial expression (quantified as the proportion of positive cells) between MLH1 and PMS2 and between MSH2 and MSH6 (Pearson r = 0.88, Spearman ρ = 0.79; and r = 0.69, ρ = 0.74; all $p < 2.2e{-}16$) (Figure S2) were consistent with known heterodimerization; correlations for other MMR proteins were less strong (Figure S2). Positive correlations between MMR proteins in stromal cells (Pearson r = 0.68–0.76, Spearman ρ = 0.70–0.78; $p < 2.2e{-}16$ all cases) suggested possible coordinate regulation (Figures S2 and S3).

AIMMeR classification of DNA MMRd versus pathologist ground truth in the SCOT trial

We examined AIMMeR performance for detection of MMRd in SCOT cases. Reasoning that MMRd should result in absent epithelial staining for one or more MMR proteins, we selected all 487 tumors in which $\geq$1 MMR protein was expressed in <20% epithelial cells. We also selected a random sample of 198 tumors from the >1,500 cases with $\geq$20% epithelial immunostaining for all MMR proteins as putative MMR proficient (MMRp) controls (Figure S4). Scanned IHC images from these 685 cases were reviewed by two expert CRC pathologists (A.E. and V.H.K.), blinded to AIMMeR results and to each other to first generate individual pathologist MMR calls and then consensus calls after discussion to resolve discrepancies. 229 cases were classified as MMRd[39] on consensus review, all of which had <20% epithelial cell expression of $\geq$1 MMR protein(s) by AIMMeR. All 198 cases in which AIMMeR detected expression of all MMR proteins in $\geq$20% epithelial cells were confirmed as MMRp. Taking the consensus pathologist calls as ground truth, calculation of AUROCs identified the lowest percentage of positive epithelial cells (calculated as the mean across TMA

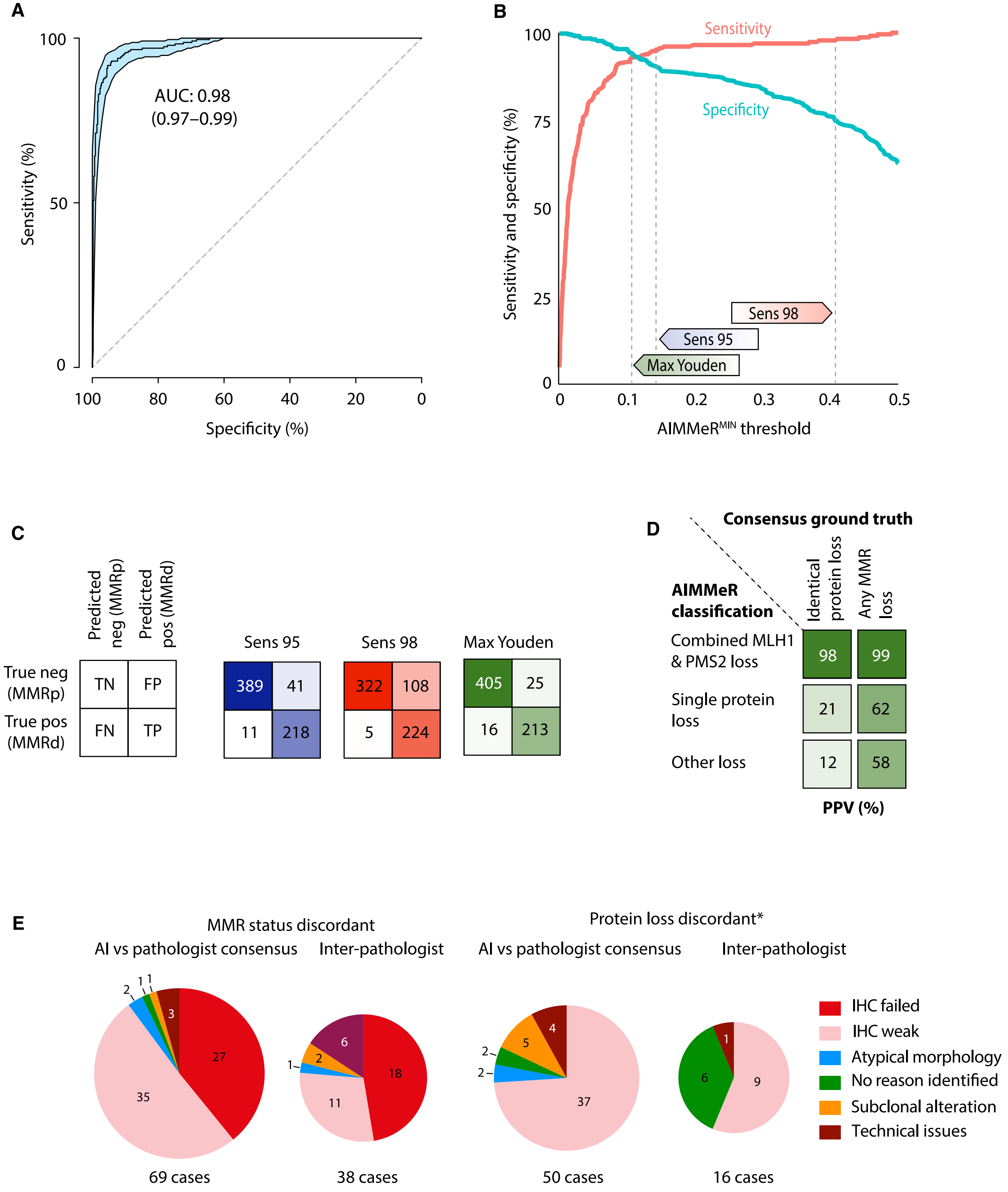

Figure 3. AIMMeR identifies mismatch repair deficiency with high accuracy in SCOT cases

(A) Receiver-operator curve (ROC) for AIMMeR classification of MMRd against consensus pathologist ground truth in 685 SCOT cases. 95% confidence intervals were obtained by bootstrap (1,000 resamples).

(legend continued on next page)

cores) among the four MMR proteins (i.e., the value from the MMR protein with the fewest positive epithelial cells) as the optimum predictor of MMRd, with AUROC of 0.98 (bootstrap 95% confidence interval [CI] = 0.97–0.99) (Figures 3A and S4). This value, henceforth referred to as AIMMeR^{MIN}, was used for the subsequent analyses.

As AUROC alone is inadequate as a measure of classification performance,[40] we determined AIMMeR sensitivity and specificity as a function of the AIMMeR^{MIN} threshold used for classification of MMR status (Figure 3B). Sensitivity increased steeply to approx. 95% at AIMMeR^{MIN} threshold of 0.15 and more modestly thereafter, while specificity decreased monotonically as a function of increasing threshold (Figure 3B). As the clinical usefulness of a test represents a trade-off between high sensitivity (few false negatives) and high specificity (few false positives), we next determined AIMMeR performance at alternative AIMMeR^{MIN} thresholds (Table 1; Figure 3C). At fixed sensitivity of 95% (proposed previously to represent clinical grade performance[18,23]), AIMMeR specificity was 91%, while a fixed threshold with 98% sensitivity had specificity of 75% (Table 1; Figure 3B). The Youden index (sensitivity plus specificity) was maximized at sensitivity of 93% and specificity of 94% (Youden^{MAX}) (Figure 3B). The proportion of all cases correctly classified as negative, or "rule-out fraction," depends on the prevalence of the condition tested and will be underestimated in our ground-truth set enriched for MMRd tumors (33.4%). We therefore also estimated this across the whole SCOT cohort after classifying the 1,330 unreviewed cases with AIMMeR^{MIN} as MMRp (based on the perfect correspondence in the consensus review set), resulting in MMRd prevalence of 11.5%. At a fixed sensitivity of 95% the rule-out fractions in the ground-truth set and whole cohorts were 0.59 and 0.86, respectively; the corresponding proportions at fixed sensitivity of 0.98 were 0.49 and 0.83 and at Youden^{MAX} 0.62 and 0.87 (Table 1).

AIMMeR classification (using the Youden^{MAX} threshold) showed substantial or better concordance with individual and consensus pathologist review at the level of both MMR status (κ = 0.79–0.82; Gwet AC1 = 0.85–0.89) and individual MMR protein loss (κ = 0.66–0.69; Gwet AC1 = 0.79–0.82) (Table S2; Figure S5). Analysis at the individual protein level also revealed notable variation in AIMMeR positive predictive value (PPV) according to the pattern of loss: combined MLH1-PMS2 loss had PPV of 99% for MMRd and 98% for combined MLH1-PMS2 loss on consensus review, while corresponding PPVs for single protein loss or other patterns were lower, possibly owing to a greater probability that they reflect artifactual staining (Figure 3D; Tables S3 and S4; Figures S5–S7). The most common cause of incorrect AIMMeR classification was inadequate immunostaining, which accounted for 90% of discordances in MMR status and 74% discordances in individual protein loss (this also accounted for most discrepancies between pathologists), with other causes including technical issues (e.g., tissue folding) and, rarely, atypical epithelial morphology (Figure 3E, Tables S4 and S5; Figure S8).

AIMMeR classification versus consensus pathologist MMR status and MSI ground truth in the QUASAR2 trial

We sought to validate AIMMeR performance for MMRd detection in the QUASAR2 trial cohort for which MSI status has also previously been determined.[38] After immunostaining and exclusion of cases failing QC and those lacking MSI data, 965 cases were informative for analysis (Figure 1B). From these, microsatellite stable (MSS) and unstable (MSI) cases with AIMMeR^{MIN} of both <20% and $\geq$20% were selected for pathologist review to generate a set of 381 cases with both pathologist MMR status and MSI ground truth (STAR Methods). Benchmarking of AIMMeR against consensus MMR calls in this set revealed an AUROC of 0.98 (bootstrap 95% CI = 0.96–0.99) (Figure 4A), and similar sensitivity across AIMMeR^{MIN} thresholds to that observed in the SCOT cohort, although specificity was lower (Figure 4B). Interestingly, AIMMeR AUROC against MSI status in these 381 cases was lower at 0.87 (95% CI = 0.83–0.92), and similar to AIMMeR AUROC against MSI status in the whole cohort (0.86, 95% CI = 0.82–0.90) (Figure 4C). Classification of MMR status using the AIMMeR^{MIN} threshold with 95% sensitivity in the SCOT training cohort had sensitivity of 99% and specificity of 66% against ground-truth MMR calls in the QUASAR2 validation cohort; corresponding values for the AIMMeR threshold with 98% sensitivity in SCOT were 100% and 48%, respectively (Table 1; Figure 4D). Thresholds defined within the QUASAR2 cohort had substantially improved specificity (and thus fewer false-positive calls) at the expense of slightly reduced sensitivity (Table 1; Figure 4D). As in SCOT, AIMMeR classification of combined MLH1-PMS2 loss had excellent PPV for both MMR loss (98%) and combined MLH1-PMS2 loss (98%) on ground-truth review, though PPV of other protein combinations was again lower (Figure 4E). We further explored the reasons for the discordant AIMMeR performance when benchmarked against either pathologist ground-truth MMR calls or MSI status (Figures 4A, 4C, and 4D) in the 381 tumors with consensus pathologist review, which included 44 of the 148 cases in which AIMMeR classification and MSI status were discordant across the whole cohort (Figure 4F). Of 20 MSS cases classified as MMRd by AIMMeR, 6 were confirmed as MMRd, while 16 of 24 MSI cases classified as MMRp by AIMMeR were confirmed as MMRp (one case was misclassified owing to non-malignant epithelial cells in the section). Thus, while AIMMeR misclassified some cases, most of the discordance between AIMMeR and MSI calls

(B) Sensitivity and specificity according to AIMMeR^{MIN} threshold. Thresholds with sensitivity of 95% (Sens 95), 98% (Sens 98), and maximum Youden index (sensitivity plus specificity) are shown.

(C) Confusion matrices showing AIMMeR classification vs. consensus pathologist ground truth at AIMMeR^{MIN} thresholds shown in (B).

(D) Positive predictive value of AIMMeR classification of MMR protein loss for the identical combination of protein loss and for any type of MMR loss. MMR status and protein loss were classified using threshold with maximal Youden index.

(E) Reasons for discordance between AI and consensus pathologist calls identified at discrepancy review for both MMR status and protein status (*limited to cases for which MMR status was concordant). Reasons for discordance between pathologists are provided for comparison. Additional detail is provided in Tables S2–S5 and Figures S2–S7; illustrative cases are shown in Figure S8.

Table 1. AIMMeR performance in training (SCOT) and validation (QUASAR2) cohorts

Cohort/threshold		Sensitivity (recall)	Specificity	Positive predictive value (precision)	Negative predictive value: Consensus review	Negative predictive value: All cases	True-negative fraction (rule-out fraction): Consensus review	True-negative fraction (rule-out fraction): All cases	False-negative fraction: Consensus review	False-negative fraction: All cases	False-negative rate	F1 score	Fowkes-Mallows score
SCOT: AIMMeR vs. pathologist consensus ground truth (AUROC = 0.98)													
685 cases with consensus review (33.4% MMRd). 1,988 cases in total (11.5% MMRd)													
Within-cohort threshold	Sensitivity 0.95	95	91	84	97	99	0.59	0.86	0.017	0.006	0.05	0.89	0.89
	Sensitivity 0.98	98	75	68	99	100	0.49	0.83	0.007	0.002	0.02	0.80	0.81
	Max Youden	93	94	90	96	99	0.62	0.87	0.024	0.008	0.07	0.91	0.91
QUASAR2: AIMMeR vs. pathologist consensus ground truth (AUROC = 0.98)													
381 cases with consensus review (23.4% MMRd)													
External (SCOT) threshold	Sens 0.95	99	66	47	100	–	0.51	–	0.003	–	0.01	0.68	0.68
	Sens 0.98	100	48	37	100	–	0.37	–	0	–	0	0.61	0.61
	Max Youden	99	74	54	100	–	0.57	–	0.003	–	0.01	0.73	0.73
Within-cohort threshold	Sens 0.95	95	89	73	98	–	0.69	–	0.012	–	0.05	0.83	0.83
	Sens 0.98	98	77	56	99	–	0.59	–	0.005	–	0.02	0.74	0.74
	Max Youden	91	95	85	97	–	0.73	–	0.021	–	0.09	0.88	0.88

reflected underlying disagreement between MMR and MSI status, as previously reported.[41]

Combined AIMMeR and pathologist classification of MMRd shows prognostic and predictive value in SCOT trial cohort

MMRd is prognostic in stage II CRC and has been reported to predict chemotherapy sensitivity. We investigated this in SCOT by combining consensus pathologist calls for 658 cases with these with AIMMeR calls for the remaining 1,330 cases. As expected, MMRd tumors were associated with older age, female gender, and right-sided location (all $p < 0.0001$, Fisher exact test) (Table S6). They also displayed significantly denser lymphocytic infiltrate, and significantly higher tumor/stromal ratio as determined by AI-based analysis (Figure S9). Consistent with previous literature, patients with MMRd tumors had longer recurrence-free interval (univariable hazard ratio [HR] = 0.69, 95% CI = 0.50–0.96, $p = 0.027$; multivariable-adjusted HR = 0.62, 95% CI = 0.44–0.88, $p = 0.007$) (Figure 5A; Table S7). Analysis in prespecified subgroups suggested the prognostic value of MMRd was independent of patient gender and disease stage; concordant with prior results, the effect size was greater in younger patients (<70 years) and right-sided tumors,[42] although interaction tests were not significant (Figure 5B). We found no evidence that MMRd predicted differential benefit from duration of chemotherapy (Figure 5C). Analysis by chemotherapy regimen revealed patients with MMRp tumors treated with capecitabine and oxaliplatin (CAPOX) had similar rate of recurrence to those who had infusional 5-fluorouracil and oxaliplain (FOLFOX) (univariable HR = 1.05, 95% CI = 0.86–1.29, $p = 0.62$; multivariable HR = 0.95, 95% CI = 0.77–1.16, $p = 0.6$), while in contrast patients with MMRd tumors treated with FOLFOX had significantly worse outcomes (univariable HR = 1.90, 95% CI = 1.01–3.57, $p = 0.046$; multivariable HR = 2.08, 95% CI = 1.09–3.97, $p = 0.027$, $P_{INTERACTION} = 0.04$). Although intriguing, this result should be interpreted with caution as the chemotherapy regimen was chosen by the treating physician, rather than by randomization.

DISCUSSION

We developed AIMMeR—a method to identify loss of DNA MMR in cancer at the single-cell level, with an AUROC of 0.98 vs. consensus pathologist ground truth in two large CRC cohorts and superior specificity to previous DL models at higher sensitivity.[18,23,27] Our analyses suggest that in unselected early-stage CRC AIMMeR used at a sensitivity of 0.98 could classify more than three-quarters of cases as MMRp, while its near-perfect ability to identify combined MLH1-PMS2 loss could potentially action reflex *BRAF* mutation or MLH1 promoter methylation testing to differentiate germline and sporadic causes[30,31] without delay caused by human review. Thus, while additional testing and validation are required, AIMMeR holds promise for clinical application as a tool to reduce pathologist workload and streamline diagnostic workflows (Figure 6) either as the initial MMR diagnostic or alternatively following DL methods.

As noted earlier, previous DL-based efforts to identify MMRd in CRC have largely focused on the use of H&E slide images, as these are inexpensive and routinely generated during

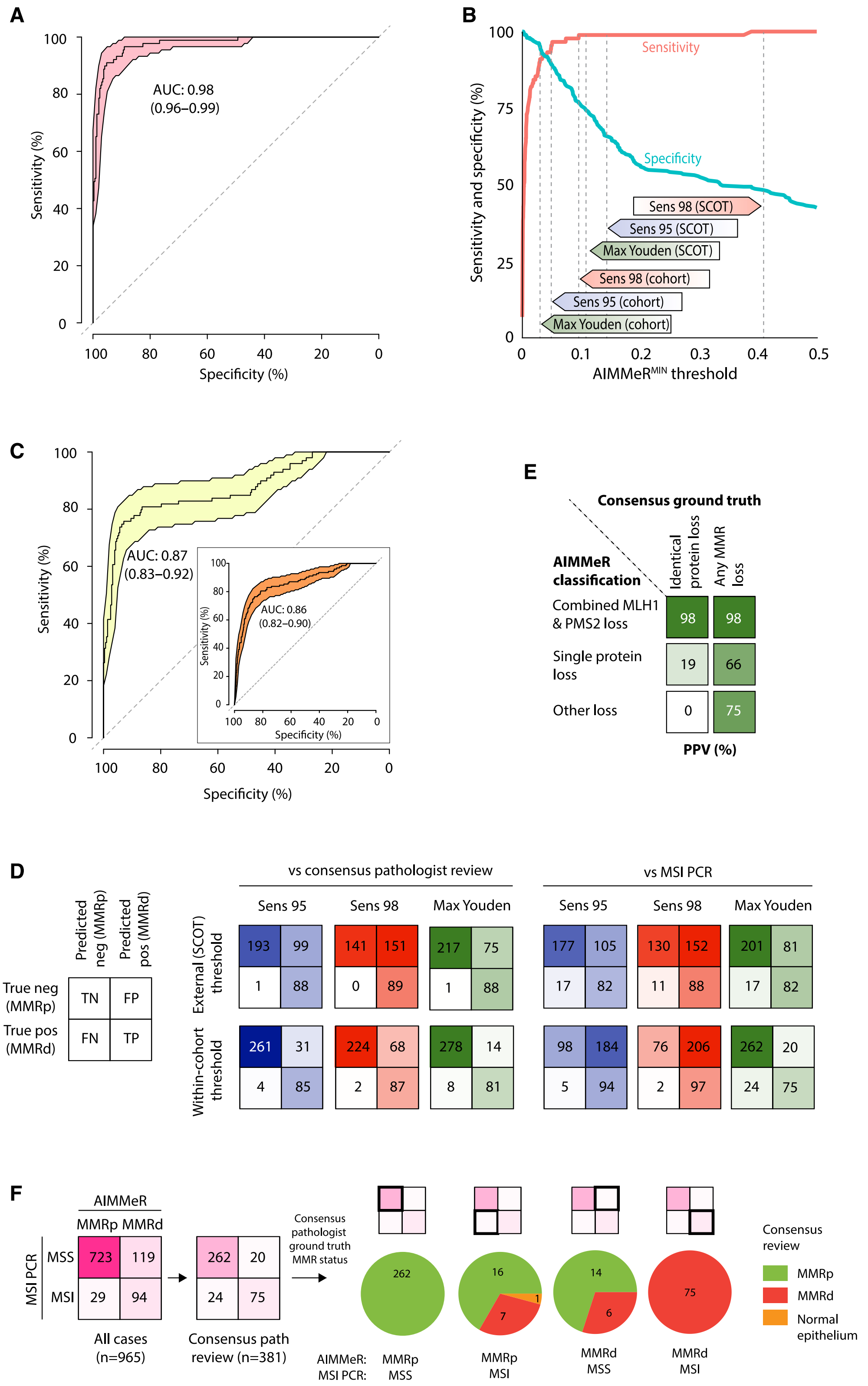

(legend on next page)

diagnosis.[17,18,23,43] DL methods using images from resection specimens[17,18,23,27] and more recently biopsies[29] have been proposed as "rule-out tests" to avoid the need for MMR IHC and pathologist review or MSI testing in predicted MMRp CRC.[23] However, using the current state of the art, roughly half of cases still require further testing (IHC or MSI) and pathologist review.[18,23,27,29] While AIMMeR requires MMR immunostaining of all tumors, this is inexpensive[44] and routine in the UK and elsewhere in accordance with clinical guidelines.[14–16] Detailed cost comparison of methods was beyond the scope of this study but is planned alongside future validation work.

While MMRd prognostic value in CRC[6,7] and variation by sidedness[42] has been demonstrated previously, our study strengthens the evidence this holds in patients treated with oxaliplatin.[9,42,45] It is also the first study to show that MMRd tumors have similar outcomes whether treated with 3 or 6 months of such treatment, although our study was only powered to detect a large difference between these groups (see STAR Methods). While the statistically significant interaction we found between MMRd and chemotherapy regimen (CAPOX vs. FOLFOX) is intriguing, this must be interpreted cautiously. Choice of chemotherapy regimen in the SCOT trial was not randomized but was rather at the discretion of the treating physician, and the similar recurrence rates between MMRp and MMRd patients treated with FOLFOX are discordant with previous results.[45] Firm conclusions regarding the relationship between MMR status and duration and type of adjuvant chemotherapy in CRC await results of adequately powered pooled analyses of multiple studies.

Strengths of our study include its large size, homogeneous patient cohorts of stage II/III CRC, high-quality curated demographic, pathological and outcome data, and multiple recruiting sites. The latter is especially relevant, as the performance of AI methods for clinical image analysis often varies between single centers, possibly owing to different protocols for sample fixation, processing, and other factors.[46] Limitations of our study are discussed in the following.

In conclusion, we developed AIMMeR—a single-cell method to identify MMRd in CRC with an AUROC of 0.98 in two independent CRC trial cohorts, enabling us to confirm MMRd prognostic and predictive value in oxaliplatin-treated patients in the SCOT study. Our study extends the potential applications of AI in CRC diagnostic pathology and holds promise for clinical implementation.

Limitations of the study

Our study has limitations. For logistical reasons, we used images from MMR immunostaining of TMA cores rather than whole slides, and it will be important to evaluate performance in these in future work. Similarly, while AIMMeR AUROC against pathologist ground truth varied only slightly depending on which TMA core was used, formal confirmation in diagnostic biopsies is planned as neoadjuvant approaches are becoming more widespread.[12,13,47] Failed or weak immunostaining caused AIMMeR to misclassify cases, as it does expert pathologists, and improving pre-analytics is a focus of our current work. Extending the results we obtained from our homogeneous trial cohorts to non-trial populations will add value and is planned. Finally, the absence of germline or somatic MMR gene sequencing meant we were unable to determine the cause of single MMR protein loss in cases where this was found.

CONSORTIA

The TransSCOT Trial Management Group includes the following (alphabetical order):

David Church, Enric Domingo, Joanne Edwards, Bengt Glimelius, Ismail Gogenur, Andrea Harkin, Jen Hay, Timothy Iveson, Emma Jaeger, Caroline Kelly, Rachel Kerr, Noori Maka, Hannah Morgan, Karin Oien, Clare Orange, Claire Palles, Campbell Roxburgh, Owen Sansom, Mark Saunders, and Ian Tomlinson. See Document S2 for consortium member affiliations.

RESOURCE AVAILABILITY

Lead contact

Further information and requests for resources and reagents should be directed to and will be fulfilled by the lead contact, David Church (david.church@well.ox.ac.uk).

Materials availability

This study did not generate new unique reagents.

Data and code availability

- Non-identifiable data reported in this paper will be shared by the lead author upon request subject to ethical restrictions and approval by the SCOT and/or QUASAR2 trial management groups where required.
- This paper does not report original code.
- Any additional information required to reanalyze the data reported in this paper is available from the lead contact on request.

Figure 4. AIMMeR identifies mismatch repair deficiency with high accuracy in QUASAR2 cases

(A) Receiver-operator curve (ROC) for AIMMeR classification of MMRd against consensus pathologist ground truth in 381 QUASAR2 cases. 95% confidence intervals were obtained by bootstrap (1,000 resamples).

(B) Sensitivity and specificity against consensus pathologist ground truth according to AIMMeR^{MIN} threshold. Thresholds with sensitivity of 95% (Sens 95), 98% (Sens 98), and maximum Youden index in the SCOT cohort, and the corresponding thresholds determined within the QUASAR2 cohort are shown.

(C) ROC for AIMMeR classification of MMR status against MSI PCR ground truth. Main panel shows curve in the 381 cases shown in (A), while inset shows that from analysis of all 965 cases with available MSI status.

(D) Confusion matrices showing AIMMeR MMR classification against consensus pathologist ground truth and against MSI PCR ground truth at AIMMeR^{MIN} thresholds defined in the SCOT cohort and QUASAR2 cohort shown in (B).

(E) Positive predictive value of AIMMeR classification of MMR protein loss for the identical combination of protein loss and for any type of MMR loss. MMR status and protein loss were classified using threshold with maximal Youden index in QUASAR2 cohort.

(F) Discordance between AIMMeR MMR classification, MSI testing, and consensus pathology MMR status. Confusion matrices of AIMMeR MMR classification against MSI PCR are shown for all 965 cases and the 381 with consensus pathologist review; pie charts show MMR status from consensus pathologist review for the four groups. Pathologist review revealed misclassification of one case in the MMRp, MSI subgroup was due to signal from non-malignant epithelial cells present in the section.

CellPress
OPEN ACCESS

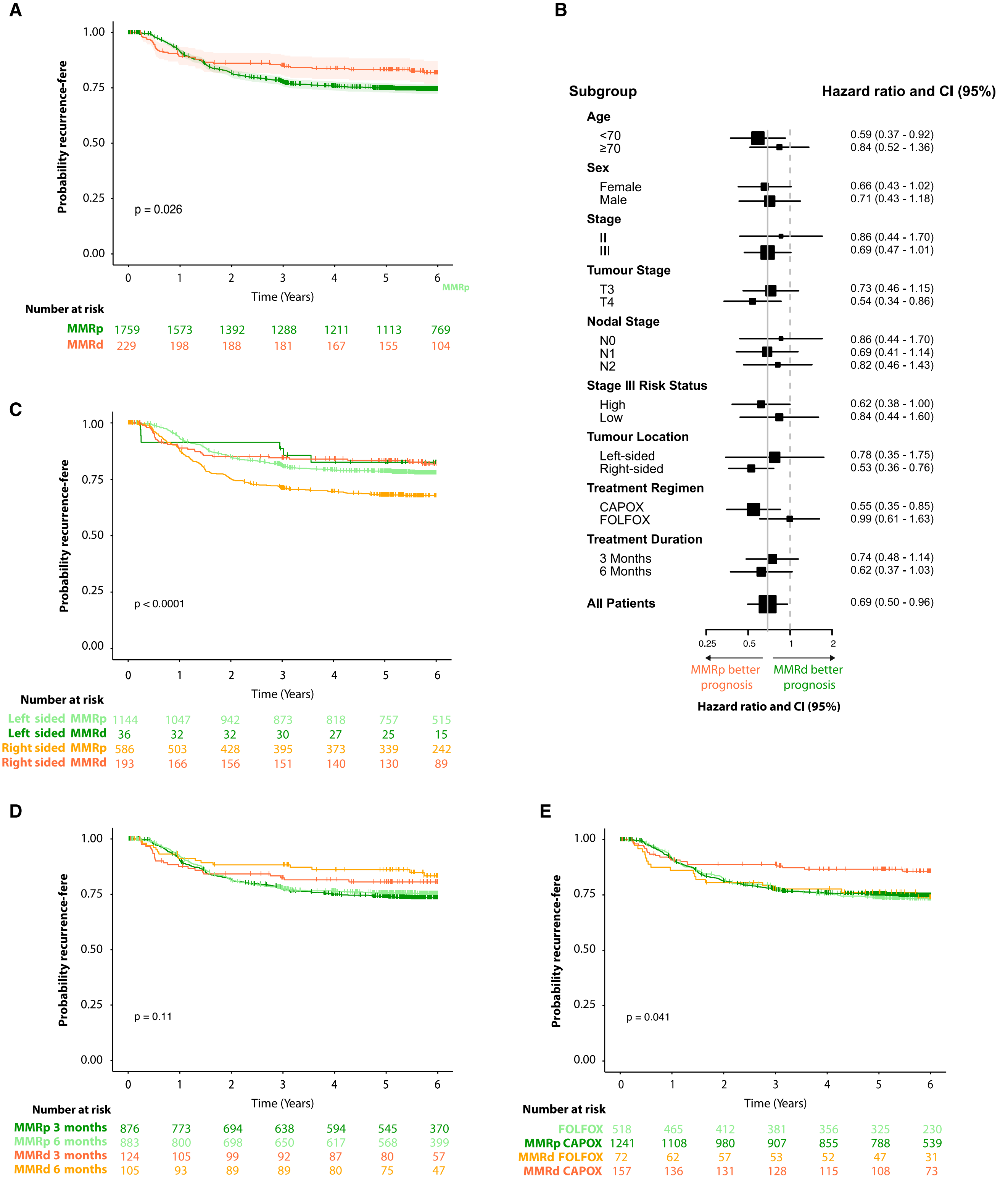

Figure 5. Prognostic and predictive value of combined AIMMeR and pathologist classification of MMRd in the SCOT trial cohort

(A) Kaplan-Meier plot showing recurrence-free interval (RFI) for patients according to MMR status.

(B) Forest plot showing hazard ratios (HR) with 95% confidence intervals (95% CI) for RFI according to MMR status within clinical and pathological subgroups by multivariable analysis*.

(legend continued on next page)

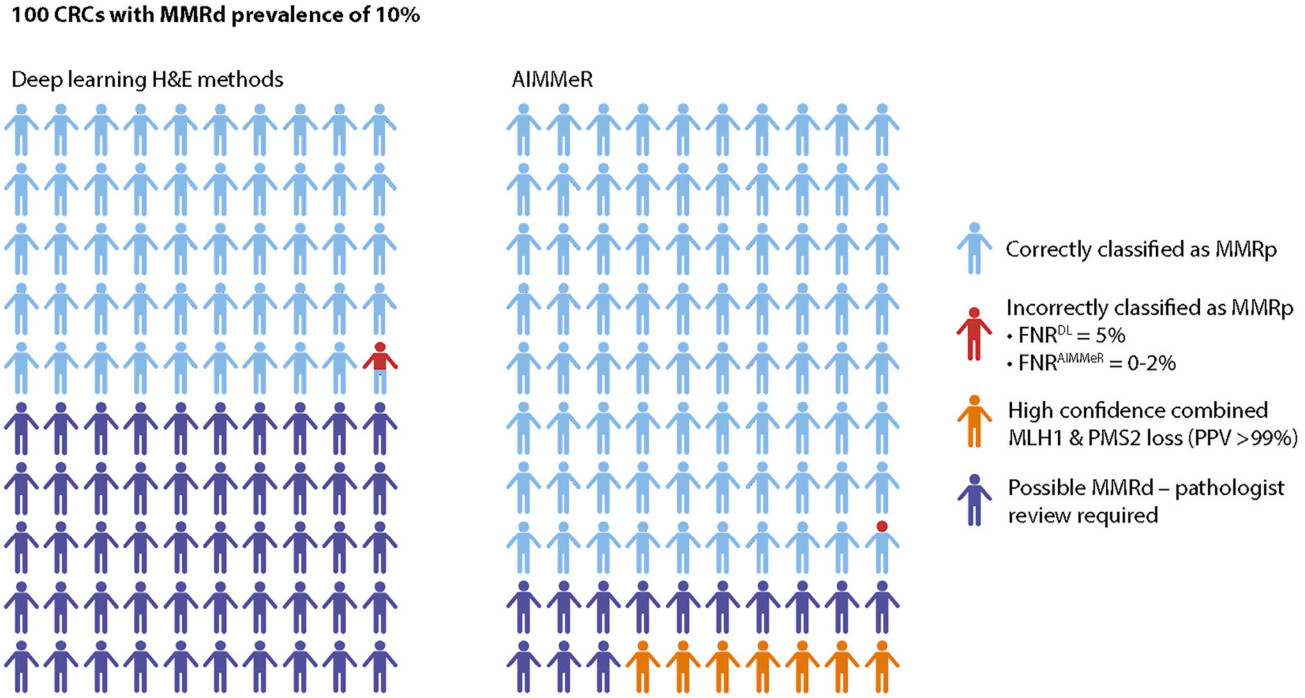

Figure 6. Potential role of AIMMeR in classification of MMR status in CRC

Plots show outcomes of application of DL methods to H&E-stained slides and of AIMMeR to IHC-stained slides in 100 colorectal cancers with MMRd prevalence of 10%. Current performance of DL methods correctly identifies 49 cases as MMRp, though current false-negative rate of 5%[23,27] (using within-cohort thresholds) means 0.5 MMRd case is misclassified as MMRp. 50 cases require further testing such as IHC with all requiring pathologist review. AIMMeR requires immunostaining of all cases but correctly identifies nearly 80% of cases as MMRp with false-negative rate of ≤2% (using within-cohort threshold) and also identifies 7 MMRd cases with combined MLH1-PMS2 loss with PPV of 98%, potentially allowing reflex testing for *BRAF* mutation or MLH1 promoter methylation. Pathologist review of cases with possible MMRd is required in 13 cases.

ACKNOWLEDGMENTS

This study was funded by the Oxford NIHR Comprehensive Biomedical Research Centre (BRC), a Cancer Research UK Advanced Clinician Scientist Fellowship (C26642/A27963) to D.N.C., and a CRUK award A25142 to the CRUK Glasgow Centre. V.H.K. acknowledges funding by the Promedica Foundation (F-87701-41-01). The views expressed are those of the authors and not necessarily those of the NHS, the NIHR, and the Department of Health.

We would like to thank the patients who participated in the SCOT and QUASAR2 trials and consented for their samples to be used for correlative research, as well as the recruiting clinicians and study teams. We are also grateful to NHSGGC for performing immunostaining and GTRF (Glasgow University) for TMA construction and scanning.

AUTHOR CONTRIBUTIONS

Conceptualization: M.N., D.N.C., and V.H.K. Data curation: M.N., F.J., A.-K.R., L.G., T.T., A.H., T.I., M.S., R.K., K.O., N.M., J.H., F.P., and E.D. Formal analysis: M.N., F.J., L.G., T.T., A.E., E.D., D.N.C., and V.H.K. Funding acquisition: I.T., O.S., V.H.K., and D.N.C. Investigation: M.N., F.J., D.N.C., and V.H.K. Methodology: M.N., V.H.K., and D.N.C. Project administration: J.H., V.H.K., and D.N.C. Resources: A.-K.R., A.H., T.I., M.S., R.K., K.O., N.M., J.H., J.E., I.T., O.S., C.K., F.P., R.K., D.K., TransSCOT group, and D.N.C. Software: M.N. and V.H.K. Supervision: V.H.K. and D.N.C. Validation: M.N., V.H.K., and D.N.C. Visualization: M.N., F.J., E.D., V.H.K., and D.N.C. Writing – original draft: D.N.C. Writing – review and editing: all authors.

DECLARATION OF INTERESTS

D.N.C. has participated in advisory boards for MSD and has received research funding on behalf of the TransSCOT consortium from HalioDx for analyses independent of this study. V.H.K. has served as an invited speaker on behalf of Indica Labs, SPCC, and Takeda and has received project-based research funding from The Image Analysis Group and Roche outside of the submitted work.

STAR★METHODS

Detailed methods are provided in the online version of this paper and include the following:

- KEY RESOURCES TABLE
- EXPERIMENTAL MODEL AND SUBJECT DETAILS
  - Ethical approval and consent to participate

(C) Kaplan-Meier plot showing RFI according tumor sidedness and MMR status.
(D) Kaplan-Meier plot showing RFI according to duration of chemotherapy and MMR status.
(E) Kaplan-Meier plot showing RFI according to chemotherapy regimen and MMR status. *p* values in (A, C, D, and E) were obtained by log rank test. Hazard ratios (HRs) in (B) were obtained by multivariable Cox proportional hazards models including prespecified covariables of age, gender, pN stage, pT stage, sidedness, treatment regimen, and treatment duration. Additional detail is provided in Tables S6 and S7; relationship between MMR status and tumor lymphocytic infiltrate and stroma is shown in Figure S9.

CellPress
OPEN ACCESS

- METHOD DETAILS
  - Tissue microarrays and immunohistochemistry
  - Microsatellite instability testing
  - Artificial intelligence-based single cell classification and mismatch repair protein quantification
  - Pathologist review of cases
  - Data processing and analysis of AIMMeR performance
- QUANTIFICATION AND STATISTICAL ANALYSIS
- ADDITIONAL RESOURCES

SUPPLEMENTAL INFORMATION

Supplemental information can be found online at https://doi.org/10.1016/j.xcrm.2024.101727.

Received: March 16, 2023
Revised: May 16, 2024
Accepted: August 15, 2024
Published: September 17, 2024

REFERENCES

1. Sung, H., Ferlay, J., Siegel, R.L., Laversanne, M., Soerjomataram, I., Jemal, A., and Bray, F. (2021). Global Cancer Statistics 2020: GLOBOCAN Estimates of Incidence and Mortality Worldwide for 36 Cancers in 185 Countries. CA A Cancer J. Clin. *71*, 209–249. https://doi.org/10.3322/caac.21660.
2. Sinicrope, F.A. (2018). Lynch Syndrome-Associated Colorectal Cancer. N. Engl. J. Med. *379*, 764–773. https://doi.org/10.1056/NEJMcp1714533.
3. Haraldsdottir, S., Hampel, H., Tomsic, J., Frankel, W.L., Pearlman, R., de la Chapelle, A., and Pritchard, C.C. (2014). Colon and Endometrial Cancers with Mismatch Repair Deficiency can Arise from Somatic, Rather Than Germline, Mutations. Gastroenterology *147*, 1308–1316.e1. https://doi.org/10.1053/j.gastro.2014.08.041.
4. Mensenkamp, A.R., Vogelaar, I.P., van Zelst-Stams, W.A., Goossens, M., Ouchene, H., Hendriks-Cornelissen, S.J., Kwint, M.P., Hoogerbrugge, N., Nagtegaal, I.D., and Ligtenberg, M.J. (2014). Somatic mutations in MLH1 and MSH2 are a frequent cause of mismatch-repair deficiency in Lynch syndrome-like tumors. Gastroenterology *146*, 643–646.e648. https://doi.org/10.1053/j.gastro.2013.12.002.
5. Kane, M.F., Loda, M., Gaida, G.M., Lipman, J., Mishra, R., Goldman, H., Jessup, J.M., and Kolodner, R. (1997). Methylation of the hMLH1 promoter correlates with lack of expression of hMLH1 in sporadic colon tumors and mismatch repair-defective human tumor cell lines. Cancer Res. *57*, 808–811.
6. Hutchins, G., Southward, K., Handley, K., Magill, L., Beaumont, C., Stahlschmidt, J., Richman, S., Chambers, P., Seymour, M., Kerr, D., et al. (2011). Value of mismatch repair, KRAS, and BRAF mutations in predicting recurrence and benefits from chemotherapy in colorectal cancer. J. Clin. Oncol. *29*, 1261–1270. https://doi.org/10.1200/JCO.2010.30.1366.
7. Bertagnolli, M.M., Redston, M., Compton, C.C., Niedzwiecki, D., Mayer, R.J., Goldberg, R.M., Colacchio, T.A., Saltz, L.B., and Warren, R.S. (2011). Microsatellite instability and loss of heterozygosity at chromosomal location 18q: prospective evaluation of biomarkers for stages II and III colon cancer–a study of CALGB 9581 and 89803. J. Clin. Oncol. *29*, 3153–3162. https://doi.org/10.1200/JCO.2010.33.0092.
8. Vilar, E., and Gruber, S.B. (2010). Microsatellite instability in colorectal cancer—the stable evidence. Nat. Rev. Clin. Oncol. *7*, 153–162. https://doi.org/10.1038/nrclinonc.2009.237.
9. Cohen, R., Taieb, J., Fiskum, J., Yothers, G., Goldberg, R., Yoshino, T., Alberts, S., Allegra, C., de Gramont, A., Seitz, J.F., et al. (2021). Microsatellite Instability in Patients With Stage III Colon Cancer Receiving Fluoropyrimidine With or Without Oxaliplatin: An ACCENT Pooled Analysis of 12 Adjuvant Trials. J. Clin. Oncol. *39*, 642–651. https://doi.org/10.1200/jco.20.01600.
10. Le, D.T., Uram, J.N., Wang, H., Bartlett, B.R., Kemberling, H., Eyring, A.D., Skora, A.D., Luber, B.S., Azad, N.S., Laheru, D., et al. (2015). PD-1 Blockade in Tumors with Mismatch-Repair Deficiency. N. Engl. J. Med. *372*, 2509–2520. https://doi.org/10.1056/NEJMoa1500596.
11. Overman, M.J., Lonardi, S., Wong, K.Y.M., Lenz, H.J., Gelsomino, F., Aglietta, M., Morse, M.A., Van Cutsem, E., McDermott, R., Hill, A., et al. (2018). Durable Clinical Benefit With Nivolumab Plus Ipilimumab in DNA Mismatch Repair-Deficient/Microsatellite Instability-High Metastatic Colorectal Cancer. J. Clin. Oncol. *36*, 773–779. https://doi.org/10.1200/jco.2017.76.9901.
12. Chalabi, M., Fanchi, L.F., Dijkstra, K.K., Van den Berg, J.G., Aalbers, A.G., Sikorska, K., Lopez-Yurda, M., Grootscholten, C., Beets, G.L., Snaebjornsson, P., et al. (2020). Neoadjuvant immunotherapy leads to pathological responses in MMR-proficient and MMR-deficient early-stage colon cancers. Nat. Med. *26*, 566–576. https://doi.org/10.1038/s41591-020-0805-8.
13. Chalabi, M.V.,Y.L., van den Berg, J., Sikorska, K., Beets, G., Lent, A.V., Grootscholten, M.C., Aalbers, A., Buller, N., Marsman, H., Hendriks, E., et al. (2022). LBA7 - Neoadjuvant immune checkpoint inhibition in locally advanced MMR-deficient colon cancer: The NICHE-2 study. Ann. Oncol. *33*, S808–S869. https://doi.org/10.1016/annonc/annonc1089.
14. Argilés, G., Tabernero, J., Labianca, R., Hochhauser, D., Salazar, R., Iveson, T., Laurent-Puig, P., Quirke, P., Yoshino, T., Taieb, J., et al. (2020). Localised colon cancer: ESMO Clinical Practice Guidelines for diagnosis, treatment and follow-up. Ann. Oncol. *31*, 1291–1305. https://doi.org/10.1016/j.annonc.2020.06.022.
15. Network, N.C.C. (2022). Colon Cancer – Version 2.2022. https://www.nccn.org/professionals/physician_gls/pdf/colon.pdf.
16. Bartley, A.N., Mills, A.M., Konnick, E., Overman, M., Ventura, C.B., Souter, L., Colasacco, C., Stadler, Z.K., Kerr, S., Howitt, B.E., et al. (2022). Mismatch Repair and Microsatellite Instability Testing for Immune Checkpoint Inhibitor Therapy: Guideline From the College of American Pathologists in Collaboration With the Association for Molecular Pathology and Fight Colorectal Cancer. Arch. Pathol. Lab Med. *146*, 1194–1210. https://doi.org/10.5858/arpa.2021-0632-CP.
17. Kather, J.N., Pearson, A.T., Halama, N., Jäger, D., Krause, J., Loosen, S.H., Marx, A., Boor, P., Tacke, F., Neumann, U.P., et al. (2019). Deep learning can predict microsatellite instability directly from histology in gastrointestinal cancer. Nat. Med. *25*, 1054–1056. https://doi.org/10.1038/s41591-019-0462-y.
18. Echle, A., Grabsch, H.I., Quirke, P., van den Brandt, P.A., West, N.P., Hutchins, G.G.A., Heij, L.R., Tan, X., Richman, S.D., Krause, J., et al. (2020). Clinical-Grade Detection of Microsatellite Instability in Colorectal Tumors by Deep Learning. Gastroenterology *159*, 1406–1416.e11. https://doi.org/10.1053/j.gastro.2020.06.021.
19. Cao, R., Yang, F., Ma, S.C., Liu, L., Zhao, Y., Li, Y., Wu, D.H., Wang, T., Lu, W.J., Cai, W.J., et al. (2020). Development and interpretation of a pathomics-based model for the prediction of microsatellite instability in Colorectal Cancer. Theranostics *10*, 11080–11091. https://doi.org/10.7150/thno.49864.
20. Lee, S.H., Song, I.H., and Jang, H.J. (2021). Feasibility of deep learning-based fully automated classification of microsatellite instability in tissue slides of colorectal cancer. Int. J. Cancer *149*, 728–740. https://doi.org/10.1002/ijc.33599.
21. Yamashita, R., Long, J., Longacre, T., Peng, L., Berry, G., Martin, B., Higgins, J., Rubin, D.L., and Shen, J. (2021). Deep learning model for the prediction of microsatellite instability in colorectal cancer: a diagnostic study. Lancet Oncol. *22*, 132–141. https://doi.org/10.1016/s1470-2045(20)30535-0.
22. Bilal, M., Raza, S.E.A., Azam, A., Graham, S., Ilyas, M., Cree, I.A., Snead, D., Minhas, F., and Rajpoot, N.M. (2021). Development and validation of a weakly supervised deep learning framework to predict the status of

molecular pathways and key mutations in colorectal cancer from routine histology images: a retrospective study. Lancet. Digit. Health *3*, e763–e772. https://doi.org/10.1016/s2589-7500(21)00180-1.

23. Echle, A., Ghaffari Laleh, N., Quirke, P., Grabsch, H.I., Muti, H.S., Saldanha, O.L., Brockmoeller, S.F., van den Brandt, P.A., Hutchins, G.G.A., Richman, S.D., et al. (2022). Artificial intelligence for detection of microsatellite instability in colorectal cancer-a multicentric analysis of a pre-screening tool for clinical application. ESMO Open *7*, 100400. https://doi.org/10.1016/j.esmoop.2022.100400.

24. Schrammen, P.L., Ghaffari Laleh, N., Echle, A., Truhn, D., Schulz, V., Brinker, T.J., Brenner, H., Chang-Claude, J., Alwers, E., Brobeil, A., et al. (2022). Weakly supervised annotation-free cancer detection and prediction of genotype in routine histopathology. J. Pathol. *256*, 50–60. https://doi.org/10.1002/path.5800.

25. Arslan, S., Mehrotra, D., Schmidt, J., Geraldes, A., Singhal, S., Hense, J., Li, X., Bass, C., Kather, J.N., Pandya, P., and Raharja-Liu, P. (2022). Deep learning can predict multi-omic biomarkers from routine pathology images: A systematic large-scale study. Preprint at bioRxiv. https://doi.org/10.1101/2022.01.21.477189.

26. Saillard, C., Dubois, R., Tchita, O., Loiseau, N., Garcia, T., Adriansen, A., Carpentier, S., Reyre, J., Enea, D., von Loga, K., et al. (2023). Validation of MSIntuit as an AI-based pre-screening tool for MSI detection from colorectal cancer histology slides. Nat. Commun. *14*, 6695. https://doi.org/10.1038/s41467-023-42453-6.

27. Niehues, J.M., Quirke, P., West, N.P., Grabsch, H.I., van Treeck, M., Schirris, Y., Veldhuizen, G.P., Hutchins, G.G.A., Richman, S.D., Foersch, S., et al. (2023). Generalizable biomarker prediction from cancer pathology slides with self-supervised deep learning: A retrospective multi-centric study. Cell Rep. Med. *4*, 100980. https://doi.org/10.1016/j.xcrm.2023.100980.

28. Schirris, Y., Gavves, E., Nederlof, I., Horlings, H.M., and Teuwen, J. (2022). DeepSMILE: Contrastive self-supervised pre-training benefits MSI and HRD classification directly from H&E whole-slide images in colorectal and breast cancer. Med. Image Anal. *79*, 102464. https://doi.org/10.1016/j.media.2022.102464.

29. Wagner, S.J., Reisenbüchler, D., West, N.P., Niehues, J.M., Zhu, J., Foersch, S., Veldhuizen, G.P., Quirke, P., Grabsch, H.I., van den Brandt, P.A., et al. (2023). Transformer-based biomarker prediction from colorectal cancer histology: A large-scale multicentric study. Cancer Cell *41*, 1650–1661.e1654. https://doi.org/10.1016/j.ccell.2023.08.002.

30. Stoffel, E.M., Mangu, P.B., Gruber, S.B., Hamilton, S.R., Kalady, M.F., Lau, M.W.Y., Lu, K.H., Roach, N., and Limburg, P.J.American Society of Clinical Oncology; European Society of Clinical Oncology (2015). Hereditary colorectal cancer syndromes: American Society of Clinical Oncology Clinical Practice Guideline endorsement of the familial risk-colorectal cancer: European Society for Medical Oncology Clinical Practice Guidelines. J. Clin. Oncol. *33*, 209–217. https://doi.org/10.1200/jco.2014.58.1322.

31. Monahan, K.J., Bradshaw, N., Dolwani, S., Desouza, B., Dunlop, M.G., East, J.E., Ilyas, M., Kaur, A., Lalloo, F., Latchford, A., et al. (2020). Guidelines for the management of hereditary colorectal cancer from the British Society of Gastroenterology (BSG)/Association of Coloproctology of Great Britain and Ireland (ACPGBI)/United Kingdom Cancer Genetics Group (UKCGG). Gut *69*, 411–444. https://doi.org/10.1136/gutjnl-2019-319915.

32. Glaire, M., Domingo, E., Nicholson, G., Novelli, M., Lawson, K., Oukrif, D., Kidal, W., Danielsen, H.E., Kerr, R., Kerr, D.J., et al. (2018). Tumour-infiltrating CD8+ lymphocytes as a prognostic marker in colorectal cancer: A retrospective, pooled analysis of the QUASAR2 and VICTOR trials. J. Clin. Oncol. *36*, 3515.

33. Pagès, F., Mlecnik, B., Marliot, F., Bindea, G., Ou, F.S., Bifulco, C., Lugli, A., Zlobec, I., Rau, T.T., Berger, M.D., et al. (2018). International validation of the consensus Immunoscore for the classification of colon cancer: a prognostic and accuracy study. Lancet *391*, 2128–2139. https://doi.org/10.1016/s0140-6736(18)30789-x.

34. Horeweg, N., de Bruyn, M., Nout, R.A., Stelloo, E., Kedziersza, K., León-Castillo, A., Plat, A., Mertz, K.D., Osse, M., Jürgenliemk-Schulz, I.M., et al. (2020). Prognostic Integrated Image-Based Immune and Molecular Profiling in Early-Stage Endometrial Cancer. Cancer Immunol. Res. *8*, 1508–1519. https://doi.org/10.1158/2326-6066.Cir-20-0149.

35. Llosa, N.J., Cruise, M., Tam, A., Wicks, E.C., Hechenbleikner, E.M., Taube, J.M., Blosser, R.L., Fan, H., Wang, H., Luber, B.S., et al. (2015). The vigorous immune microenvironment of microsatellite instable colon cancer is balanced by multiple counter-inhibitory checkpoints. Cancer Discov. *5*, 43–51. https://doi.org/10.1158/2159-8290.Cd-14-0863.

36. Domingo, E., Freeman-Mills, L., Rayner, E., Glaire, M., Briggs, S., Vermeulen, L., Fessler, E., Medema, J.P., Boot, A., Morreau, H., et al. (2016). Somatic POLE proofreading domain mutation, immune response, and prognosis in colorectal cancer: a retrospective, pooled biomarker study. Lancet. Gastroenterol. Hepatol. *1*, 207–216. https://doi.org/10.1016/s2468-1253(16)30014-0.

37. Iveson, T.J., Kerr, R.S., Saunders, M.P., Cassidy, J., Hollander, N.H., Tabernero, J., Haydon, A., Glimelius, B., Harkin, A., Allan, K., et al. (2018). 3 versus 6 months of adjuvant oxaliplatin-fluoropyrimidine combination therapy for colorectal cancer (SCOT): an international, randomised, phase 3, non-inferiority trial. Lancet Oncol. *19*, 562–578. https://doi.org/10.1016/s1470-2045(18)30093-7.

38. Kerr, R.S., Love, S., Segelov, E., Johnstone, E., Falcon, B., Hewett, P., Weaver, A., Church, D., Scudder, C., Pearson, S., et al. (2016). Adjuvant capecitabine plus bevacizumab versus capecitabine alone in patients with colorectal cancer (QUASAR 2): an open-label, randomised phase 3 trial. Lancet Oncol. *17*, 1543–1557. https://doi.org/10.1016/s1470-2045(16)30172-3.

39. Loughrey, M.Q.,P., and Shepherd, N.A. (2018). Dataset for histopathological reporting of colorectal cancer. https://www.rcpath.org/static/c8b61ba0-ae3f-43f1-85ffd3ab9f17cfe6/G049-Dataset-for-histopathological-reporting-of-colorectal-cancer.pdf.

40. Kleppe, A. (2022). Area under the curve may hide poor generalisation to external datasets. ESMO Open *7*, 100429. https://doi.org/10.1016/j.esmoop.2022.100429.

41. Watson, N., Grieu, F., Morris, M., Harvey, J., Stewart, C., Schofield, L., Goldblatt, J., and Iacopetta, B. (2007). Heterogeneous staining for mismatch repair proteins during population-based prescreening for hereditary nonpolyposis colorectal cancer. J. Mol. Diagn. *9*, 472–478. https://doi.org/10.2353/jmoldx.2007.060162.

42. Sinicrope, F.A., Mahoney, M.R., Smyrk, T.C., Thibodeau, S.N., Warren, R.S., Bertagnolli, M.M., Nelson, G.D., Goldberg, R.M., Sargent, D.J., and Alberts, S.R. (2013). Prognostic impact of deficient DNA mismatch repair in patients with stage III colon cancer from a randomized trial of FOLFOX-based adjuvant chemotherapy. J. Clin. Oncol. *31*, 3664–3672. https://doi.org/10.1200/jco.2013.48.9591.

43. Kather, J.N., Heij, L.R., Grabsch, H.I., Loeffler, C., Echle, A., Muti, H.S., Krause, J., Niehues, J.M., Sommer, K.A.J., Bankhead, P., et al. (2020). Pan-cancer image-based detection of clinically actionable genetic alterations. Nat. Can. (Ott.) *1*, 789–799. https://doi.org/10.1038/s43018-020-0087-6.

44. Snowsill, T.M., Ryan, N.A.J., Crosbie, E.J., Frayling, I.M., Evans, D.G., and Hyde, C.J. (2019). Cost-effectiveness analysis of reflex testing for Lynch syndrome in women with endometrial cancer in the UK setting. PLoS One *14*, e0221419. https://doi.org/10.1371/journal.pone.0221419.

45. Zaanan, A., Shi, Q., Taieb, J., Alberts, S.R., Meyers, J.P., Smyrk, T.C., Julie, C., Zawadi, A., Tabernero, J., Mini, E., et al. (2018). Role of Deficient DNA Mismatch Repair Status in Patients With Stage III Colon Cancer Treated With FOLFOX Adjuvant Chemotherapy: A Pooled Analysis From 2 Randomized Clinical Trials. JAMA Oncol. *4*, 379–383. https://doi.org/10.1001/jamaoncol.2017.2899.

46. Van Eycke, Y.R., Allard, J., Salmon, I., Debeir, O., and Decaestecker, C. (2017). Image processing in digital pathology: an opportunity to solve

CellPress
OPEN ACCESS

inter-batch variability of immunohistochemical staining. Sci. Rep. *7*, 42964. https://doi.org/10.1038/srep42964.

47. Morton, D., Seymour, M., Magill, L., Handley, K., Glasbey, J., Glimelius, B., Palmer, A., Seligmann, J., Laurberg, S., Murakami, K., et al. (2023). Preoperative Chemotherapy for Operable Colon Cancer: Mature Results of an International Randomized Controlled Trial. J. Clin. Oncol. *41*, 1541–1552. https://doi.org/10.1200/jco.22.00046.
48. Sun, K.H.a.X.Z.a.S.R.a.J. (2014). Deep Residual Learning for Image Recognition.
49. Bossuyt, P.M., Reitsma, J.B., Bruns, D.E., Gatsonis, C.A., Glasziou, P.P., Irwig, L., Lijmer, J.G., Moher, D., Rennie, D., de Vet, H.C.W., et al. (2015). STARD 2015: an updated list of essential items for reporting diagnostic accuracy studies. Br. Med. J. *351*, h5527. https://doi.org/10.1136/bmj.h5527.
50. McShane, L.M., Altman, D.G., Sauerbrei, W., Taube, S.E., Gion, M., and Clark, G.M.; Statistics Subcommittee of the NCI-EORTC Working Group on Cancer Diagnostics (2005). REporting recommendations for tumour MARKer prognostic studies (REMARK). Br. J. Cancer *93*, 387–391. https://doi.org/10.1038/sj.bjc.6602678.
51. Royston, P., and Parmar, M.K.B. (2016). Augmenting the logrank test in the design of clinical trials in which non-proportional hazards of the treatment effect may be anticipated. BMC Med. Res. Methodol. *16*, 16. https://doi.org/10.1186/s12874-016-0110-x.
52. Freidlin, B., and Korn, E.L. (2019). Methods for Accommodating Nonproportional Hazards in Clinical Trials: Ready for the Primary Analysis? J. Clin. Oncol. *37*, 3455–3459. https://doi.org/10.1200/jco.19.01681.

STAR★METHODS

KEY RESOURCES TABLE

REAGENT or RESOURCE	SOURCE	IDENTIFIER
Antibodies		
MLH1 clone ES05 (1:100)	Leica	Lot no. 6063898 (RRID:AB_1055422)
MSH2 clone 79H11 (undiluted)	Leica	Lot no. 72212
MSH6 clone EP49 (1:80)	Dako	Cat no. 1164717 (RRID:AB_2889975)
PMS2 clone EP51 (1:50)	Dako	Cat no. 1160500 (RRID:AB_3331634)
Biological samples		
Tumor samples from SCOT trial	SCOT trial (ISRCTN59757862)	Iveson et al.[37]
Tumor samples from QUASAR2 trial	QUASAR2 trial (ISRCTN45133151)	Kerr et al.[38]
Software and algorithms		
R version 4.2.2 (2022-10-31)	Comprehensive R Network	https://www.r-project.org/
Tidyverse version 1.3.2		https://www.tidyverse.org
ggplot2 version 3.4.0		https://ggplot2.tidyverse.org
ggpubr version 0.4.0		https://cran.r-project.org/web/packages/ggpubr/index.html
cowplot version 1.1.1		https://cran.r-project.org/web/packages/cowplot/index.html
stringr version 1.4.1		https://cran.r-project.org/web/packages/stringr/index.html
riverplot version 0.1.0		https://cran.r-project.org/src/contrib/Archive/riverplot/
corrplot version 0.9.2		https://cran.r-project.org/web/packages/corrplot/corrplot.pdf
ggcorrplot version 0.1.4		https://cran.r-project.org/web/packages/ggcorrplot/ggcorrplot.pdf
irr version 0.84.1		https://cran.r-project.org/web/packages/irr/index.html
irrCAC version 1.0		https://cran.r-project.org/web/packages/irrCAC/index.html
survminer version 0.4.9.oc		https://cran.r-project.org/web/packages/survminer/index.html
Other		
Hamamatsu NanoZoomer	Hamamatsu	N/A
NVIDIA GeForce RTX 2080 Ti 11 GB	NVIDIA	N/A

EXPERIMENTAL MODEL AND SUBJECT DETAILS

Details of the SCOT (ISRCTN59757862) and QUASAR2 (ISRCTN45133151) trials have been reported previously.[37,38] SCOT compared the efficacy of 12 vs. 24 weeks of oxaliplatin-based adjuvant chemotherapy following curative-intent resection of stage III or high-risk stage II (any of: pT4 primary, tumor obstruction, <10 lymph nodes harvested, grade 3 histology, perineural/extramural venous/lymphatic invasion) colorectal cancer. 6,088 patients were randomised across 237 sites between March 2008 and November 2013. The study met its primary endpoint, with the shorter course of chemotherapy confirmed to be non-inferior (HR = 1.01, 95% CI = 0.91–1·11, test for non-inferiority $p = 0 \cdot 012$)[37] and associated with improved quality of life. QUASAR2 randomised patients of ECOG PS 0 or 1 to capecitabine or capecitabine plus bevacizumab after resection of stage III/high-risk stage II CRC between April 2005 and October 2010. Analysis of the primary endpoint of DFS demonstrated no benefit of bevacizumab. 3,076 of 6,088 patients from the SCOT trial, and 1,195 of 1,952 patients from the QUASAR2 trial consented to donate samples for research; characteristics were similar to the total study populations.[38]

CellPress
OPEN ACCESS

Ethical approval and consent to participate

Ethical approval for patient recruitment and sample collection in the SCOT and QUASAR2 trials was approved centrally and at all recruiting centers. Ethical approval for anonymized tumor molecular analysis was granted by Oxfordshire Research Ethics Committee B (Approval No 05\Q1605\66).

METHOD DETAILS

Tissue microarrays and immunohistochemistry

Tissue microarrays (TMAs) were constructed from 0.6mm punched cores from formalin-fixed paraffin embedded (FFPE) blocks following review by the TransSCOT pathologists (KO and NM). 2,352 cases with adequate tumor content (epithelial cell fraction of greater than 30%) were included in TMAs. 1,788 of these cases had two cores taken from the tumor center (TC) and two from the invasive margin (IM) (i.e., a total of four cores per case), while 564 cases had sufficient tumor for additional replicate cores to total of eight cores per case (four CT, four IM), giving a total of 23,328 cores across 2,352 cases. TMAs for the QUASAR2 trial were made following review by the study pathologist (FP) to confirm tumor cellularity. 1,195 cases were included in TMAs, each of which had 3 cores from the center of the tumor. Immunohistochemistry (IHC) for MMR proteins MLH1, MSH2, MSH6 and PMS2 in both the SCOT and QUASAR2 cohorts was performed in an accredited UK National Health Service (NHS) diagnostic pathology laboratory (Queen Elizabeth University Hospital, NHS Greater Glasgow & Clyde Trust, Glasgow, UK) to clinical standards by standard methods using ISO approved antibodies and concentrations: MLH1 clone ES05, Leica (Newcastle, UK), Lot no. 6063898, (1:100); MSH2 clone 79H11, Leica, Lot no. 72212, (undiluted); MSH6 clone EP49, Dako (Glostrup, Denmark), Cat no. 1164717, (1:80); PMS2 clone EP51, Dako– Cat no. 1160500, (1:50). Stained slides were scanned using a Hamamatsu NanoZoomer (Hamamatsu, Welwyn Garden City, UK) scanner at 40x and a resolution of 0.22 micron per pixel.

Microsatellite instability testing

Details of MSI testing in QUASAR2 samples have been reported previously.[38] DNA was extracted from 40 μm tissue scrolls cut from tumor blocks with tumor cellularity of >80% cells and microdissection of 10 μm slides for lower cellularity cases, guided by H&E section. FFPE tumor material was digested with proteinase K, and DNA was extracted with the DNeasy Kit (Qiagen, Hilden, Germany). MSI status was determined using Bethesda markers (BAT25, BAT26, D2S123, D5346, and D17S250) and BAT40, a mononucleotide repeat marker. Tumors were classified as MSI if $\geq$ 40% markers were unstable. Details of PCR primers and reaction conditions were provided previously[38]

Artificial intelligence-based single cell classification and mismatch repair protein quantification

Expression of MMR proteins was quantified on the digital TMA slides at the single nuclei level using a single consumer grade GPU (NVIDIA GeForce RTX 2080 Ti 11 GB) and HALO digital image analysis software version 3.3 (Indica Labs, Corrales, NM, USA). First, TMAs were segmented into spots corresponding to individual cores and subjected to rigorous visual quality control (QC). Spots with missing cores, damaged or insufficient tumor tissue were excluded from further analysis. For nuclear segmentation, we adapted the HALO AI nuclear Nuclei Seg model, which is based on a ResNet34 architecture with additional watershed post processing for segmenting clustered nuclei (Deep Residual Learning for Image Recognition; Kaiming He and Xiangyu Zhang and Shaoqing Ren and Jian Sun; https://arxiv.org/abs/1512.03385) and pre-trained to segment H&E− and DAB-stained nuclei on brightfield images. Additional examples of nuclei (n = 2761, total area = 825μm^2) and background (n = 352, total area = 1698μm^2) from SCOT TMAs were manually annotated and added to the training set to achieve optimal nuclear segmentation results in immunohistochemically stained tissue. Accuracy of nuclear segmentation was verified by visual pathologist review. The HALO AI v3.6.4134 Nuclei Phenotyper algorithm, based on a ResNet18 architecture[48] was then trained on SCOT TMAs to classify segmented nuclei or other objects into one of the following classes according to cell or object type and individual MMR protein expression (derived from DAB staining): (i) positive tumor cells; (ii) negative tumor cells; (iii) positive stromal cells; (iv) negative stromal cells; (v) lymphocytes (positive and negative); (vi) strong background (e.g., nuclei in out-of-focus cores or strongly DAB-stained non-nuclear objects such as tissue folds, apoptotic bodies, and cellular debris); or (vii) weak background (e.g., weakly DAB-stained objects, such as mucus or extracellular matrix components) (Figure S1A). Initial training of the Nuclei Phenotyper used approximately 21,000 annotated nuclei each from all five nuclear classes and background class (strong and weak background, cumulatively) for a total of (almost 127 000 annotated nuclei (Table S1). A held-out test set of ~9000 annotated nuclei was used to measure the accuracy of the Nuclei Phenotyper (Table S1). Mark-up images for nucleus segmentation and classification were generated, and the accuracy of nuclear classification was confirmed on the held-out test set and by pathologist review (Table S1; Figure S1B). The final combined method – AIMMeR – was used to estimate the number of cells or objects in each of the seven classes above for each of the four MMR proteins in 23,312 TMA cores from 2,352 SCOT cases after loss of 16 cores during immunostaining, and 1,195 cases in the QUASAR2 cohort. No data from the QUASAR2 cohort were used for training to ensure that this test set was completely unseen to the algorithm for performance testing.

Pathologist review of cases

Images of MMR staining from cases for pathological review from the SCOT and QUASAR2 trials were independently reviewed by two expert GI pathologists (VK and AE) blinded to the results of AIMMeR assessment and to each other. Review of SCOT cases included all 487 tumors for which AIMMeR^{MIN} was <20%, and 198 cases selected at random from 1,330 tumors in which all four MMR proteins were expressed in $\geq$20% epithelial cells in all TMA cores. Review of QUASAR2 cases included 339 (of 431) cases for which AIMMeR^{MIN} was <20% (regardless of MSI status), 17 of 18 MSI cases for which AIMMeR^{MIN} was $\geq$20% and 149 of 539 MSS cases for which AIMMeR was $\geq$20%. For each case, pathologists classified MMR status as retained or lost, and recorded the exact combination of proteins lost in the latter case. Cases with failed staining were documented as such. Pathologists also noted any unusual patterns of staining, and the presence of artifacts which could conceivably impact the performance of automated analysis. The results of individual pathologist review were then combined with each other and with the AIMMeR results, and discordances between individual pathologists and between pathologist and AIMMeR noted. For the SCOT cohort consensus pathologist ground truth was established as follows.

(1) cases where the combination of MMR protein expression was fully concordant across both pathologists and AIMMeR (e.g., classified as retained by all, or classified as combined MLH1-PMS2 loss by all), were documented as per the unanimous classification
(2) cases where the combination of MMR protein expression was discordant, either between pathologists, of between pathologists and AIMMeR results were discussed at a discrepancy meeting. At this, images were reviewed and consensus on the final ground truth classification was reached by discussion between pathologists, and the putative reason for discordance was recorded.
(3) for a subset of 84 cases consensus ground truth was taken following dual pathologist review at a discrepancy meeting without individual pathologist review beforehand. These cases were not used for determination of inter-rater reliability metrics between individual pathologists and AIMMeR results.

For the QUASAR2 trial, the consensus pathologist ground truth set included cases for which pathologist review was concordant, and excluded all discordant and failed cases. A discrepancy meeting was not held. For both cohorts, AIMMeR, individual pathologist and consensus pathologist classification were used to establish performance of AIMMeR compared with ground truth and individual pathologist review.

Data processing and analysis of AIMMeR performance

AIMMeR-derived core level data and image/case metadata were stored as a csv file and processed to obtain case-level results and summary data. Correlation between markers was evaluated by parametric Pearson r and non-parametric Spearman ρ. AIMMeR performance for detection of MMRd was determined by calculation of area under the receiver-operator curve (AUROC) and Youden index, using the consensus pathologist review as ground truth. 95% confidence intervals and out of bag estimates were obtained by bootstrap ($n = 1{,}000$). Inter-rater reliability ratings were calculated using Cohen's Kappa and Gwet AC1 for cases in which pathologist review was done. As both of these metrics are influenced by the prevalence of groups for classification, and our selection of cases for review was biased toward cases with MMRd, we calculated predicted values for analysis of the whole cohort, based on the assumption that all cases with $\geq$20% positive epithelial cells by AIMMeR would be confirmed as MMRd by pathologists (as we determined was the case for the 198 selected at random). The relationship between classification of MMR status and the combination of MMR protein expression determined by AIMMeR and by consensus pathologist review was illustrated by Sankey plots.

QUANTIFICATION AND STATISTICAL ANALYSIS

Associations between MMR status and clinicopathological characteristics of patients were determined by parametric t test and by Chi-square test in the case of continuous and categorical data respectively. Reporting of AIMMeR performance and biomarker analyses of MMRd (Table S8) were performed in accordance with the STARD[49] and REMARK[50] guidelines respectively, with checklists provided as detailed in Tables S9 and S10. The endpoint for time-to-event analyses was recurrence-free interval (RFI) of CRC, defined as the time from randomization to CRC relapse, with censoring at last contact or death in case of no recurrence. Survival curves were plotted using the Kaplan-Meier method and compared by the log rank test. Hazard ratios (HRs) were determined by univariable analysis, and by multivariable analysis adjusted for confounders using Cox proportional hazards models. Covariables for inclusion in multivariable models were prespecified, and no variable selection was performed. Inspection of scaled Schoenfeld residuals revealed violation of proportional hazards for analysis of MMR status owing to early recurrences in the MMRd group; hazard ratios should thus be interpreted in the light of this but are preferred over alternatives such as restricted mean survival time (RMST)[51] for clinical interpretability and consistency with existing literature.[52] Time to event analyses used all informative cases and excluded cases with missing data (i.e., no imputation was performed). The sample size was not pre-determined. A power calculation was performed based on 2,000 cases with 500 recurrences (i.e., similar frequency to the total trial population), assuming prevalence of MMRd or 0.1 and equal proportions of patients treated with 3 months and 6 months chemotherapy in the MMRd and MMRp groups. This demonstrated power to detect an MMRd*chemotherapy duration interaction with difference in hazard ratios of 2.3 or greater, using a

1-β of 0.8 and a two-sided αof 0.05. Sample sizes and methods used for statistical analyses are provided in the text and figure legends where reported. All statistical tests were two-sided, and hypothesis testing was performed at the 5% significance level.

ADDITIONAL RESOURCES

All analyses were performed using R (Comprehensive R Network) version 4.2.2 (2022-10-31) using R Studio version RStudio 2022.07.1, build 554. Plots were exported as vector graphics. Scanned slide images were resized and cropped in Photoshop (Adobe, San Jose CA, USA). Images and figure panels were edited in Illustrator (Adobe). R packages used in this study included: Tidyverse version 1.3.2; ggplot2 version 3.4.0; ggpubr version 0.4.0; cowplot version 1.1.1; stringr version 1.4.1; riverplot version 0.1.0; corrplot version 0.9.2; ggcorrplot version 0.1.4; irr version 0.84.1; irrCAC version 1.0; survminer version 0.4.9.oc.

Cell Reports Medicine, Volume *5*

Supplemental information

Single-cell AI-based detection and prognostic and predictive value of DNA mismatch repair deficiency in colorectal cancer

Marta Nowak, Faiz Jabbar, Ann-Katrin Rodewald, Luciana Gneo, Tijana Tomasevic, Andrea Harkin, Tim Iveson, Mark Saunders, Rachel Kerr, Karin Oein, Noori Maka, Jennifer Hay, Joanne Edwards, Ian Tomlinson, Owen Sansom, Caroline Kelly, Francesco Pezzella, David Kerr, Alistair Easton, Enric Domingo, TransSCOT group, Viktor H. Koelzer, and David N. Church

Supplemental information to Nowak et al.

Single cell AI-based detection, prognostic and predictive value of DNA mismatch repair deficiency in colorectal cancer.

Supplementary material

Contents

Table S1. Details of training and test set used for the AIMMeR development in HALO digital image analysis software v3.3. 2

Table S2. Agreement between AI and pathologist review for classification of MMR status and protein loss 3

Table S3. Classification of MMR protein loss by AI and consensus pathology review by type of MMR loss 4

Table S4. Reasons for discordance between AI and consensus pathologist classification 5

Table S5. Reasons for discordance between individual pathologist classification 6

Table S6. Clinicopathological characteristics of SCOT trial by MMR status 7

Table S7. Univariable and multivariable analysis of recurrence-free interval in SCOT trial cohort according to clinicopathologic factors and MMR status 8

Table S8. Biomarker analyses performed and reported in this study 9

Figure S1. Nuclear segmentation by object class 10

Figure S2. Scatterplots showing correlation between epithelial MMR protein expression across cases 11

Figure S3. Scatterplots showing correlation between stromal MMR protein expression across cases .. 12

Figure S4. Calculation of AUROC for alternative methods for classification of MMR loss 13

Figure S5. Relationship between AI and consensus pathologist calls for combinations of MMR protein loss 16

Figure S6. Cases discordant between AI-based and pathologist classification 17

Figure S7. Tumor lymphocytic infiltrate and tumor/stroma ratio by MMR status 18

Table S1. Details of training and test set used for the AIMMeR development in HALO digital image analysis software v3.3. Related to STAR methods

**Training Set**		
**Class**	**Objects**	**Accuracy**
positive tumor cells	31,786	NA
negative tumor cells	16,607	NA
positive stroma	20,881	NA
negative stroma	13,030	NA
lymphocytes (positive and negative)	19,404	NA
strong background	22,356	NA
weak background	2,848	NA
sum	126,912	NA
**Test Set**		
**Class**	**Object used for test**	**Accuracy**
positive tumor cells	1796	0.95
negative tumor cells	1604	0.85
positive stroma	1004	0.93
negative stroma	868	0.89
lymphocytes (positive and negative)	2471	0.98
strong and weak background	1200	0.92
sum/overall accuracy	8,943	0.92

Table S2. Agreement between AI and pathologist review for classification of MMR status and protein loss Related to Figure 3.

Group	Comparison	Number of cases	Cohen kappa (95% CI)	Gwet AC1 (95% CI)
Cases with consensus pathologist review ± blinded individual pathologist review				
MMR status (retained vs lost)	AI vs pathologist consensus	685	0.79 (0.75–0.84)	0.87 (0.84–0.90)
	AI vs pathologist 1 (AE)	601*	0.82 (0.78–0.87)	0.89 (0.86–0.92)
	AI vs pathologist 2 (VK)	601*	0.79 (0.74–0.84)	0.85 (0.82–0.89)
	Pathologist 1 vs 2	601*	0.88 (0.84–0.92)	0.92 (0.89–0.94)
Individual MMR protein loss	AI vs pathologist consensus	685	0.69 (0.65–0.73)	0.82 (0.79–0.85)
	AI vs pathologist 1 (AE)	601*	0.66 (0.62–0.71)	0.79 (0.76–0.83)
	AI vs pathologist 2 (AE)	601*	0.66 (0.62–0.71)	0.79 (0.75–0.82)
	Pathologist 1 vs 2	601*	0.84 (0.81–0.88)	0.91 (0.88–0.93)
Predicted values† in total study population				
MMR status (retained vs lost)	AI vs pathologist consensus	685 analysed 1,331 predicted†	0.85 (0.82–0.88)	0.96 (0.95–0.97)
Individual MMR protein loss	AI vs pathologist consensus	601 analysed 1,331 predicted†	0.75 (0.71–0.79)	0.94 (0.93–0.95)

Individual pathologist review of MMR status and individual MMR protein expression was performed blinded to results of AI-based analysis and the interpretation of the other pathologist. All cases with discordance between AI and one or both pathologists, as well as all cases where individual pathologists were discordant were reviewed at a discrepancy meeting, with final status resolved by discussion. *excludes subset of cases with consensus pathology review but not blinded individual pathological review. †Predicted values are calculated based on the assumption of equivalent concordance between AI and pathologist review in all 1,529 cases with ≥20% epithelial cells positive for all MMR proteins as that obtained by comparison of the randomly-selected subset of 198 cases (100% agreement).

Table S3. Classification of MMR protein loss by AI and consensus pathology review by type of MMR loss. Related to Figure 3.

AI classification		Consensus pathologist classification		PPV for MMR status	PPV for protein loss
Group	Number	Group	Number		
MLH1 & PMS2 or MSH2 & MSH6 loss	147	MLH1/PMS2 or MSH2/MSH6	140	0.966	0.952*
		single protein	2		
		Other loss	0		
		retained	2		
		fail	3		
Single MMR protein loss	89	MLH1/PMS2 or MSH2/MSH6	35	0.618	0.213†
		single protein	19		
		other loss	1		
		retained	22		
		fail	12		
Other MMR loss	26	MLH1/PMS2 or MSH2/MSH6	11	0.577	0.115‡
		single protein	1		
		other loss	3		
		retained	1		
		fail	10		
MMRp	423	MLH1/PMS2 or MSH2/MSH6	14	0.955	0.955§
		single protein	3		
		Other loss	0		
		retained	404		
		fail	2		

* denotes PPV for combined MLH1 &PMS2 loss or combined MSH2 & MSH6 loss; † denotes PPV for single MMR protein loss; ‡ denotes PPV for other patterns of MMR protein loss; § denotes PPV for retained MMR expression

Table S4. Reasons for discordance between AI and consensus pathologist classification. Related to Figure 3.

Reason for discordance	MMR status discordant		MMR status concordant but protein loss discordant	
	N	%	N	%
Immunostaining failed	27	39.1	NA	NA
Immunostaining weak/heterogenous	34	49.3	35	70.0
Background/cytoplasmic immunostaining	1	1.4	2	4.0
Technical: tissue folded	1	1.4	0	0.0
Technical: shadow/out of focus	2	2.9	4	8.0
Atypical epithelial morphology	2	2.9	2	4.0
Subclonal alteration	1	1.4	5	10.0
No reason identified	1	1.4	2	4.0

Table S5. Reasons for discordance between individual pathologist classification. Related to Figure 3.

Reason for discordance	MMR status discordant		MMR status concordant but protein loss discordant	
	N	%	N	%
Immunostaining failed	18	47.4	0	NA
Immunostaining weak/heterogenous	11	28.9	8	50
Background/cytoplasmic immunostaining	0	0	1	6.3
Technical: tissue folded	1	2.6	0	0
Technical: shadow/out of focus	3	7.9	0	0
Technical: other	2	5.3	1	6.3
Atypical epithelial morphology	1	2.6	0	0
Subclonal alteration	2	5.3	0	0
No reason identified	0	0	6	37.5

Table S6. Clinicopathological characteristics of SCOT trial by MMR status. Related to Figure 5.

	MMRp		MMRd		p-value[2]
Total	1,759		229		
Age					
Median IQR					<0.001
<70	1,302	74	138	60	
>/70	457	26	91	40	
Gender					<0.001
Female	669	38	133	58	
Male	1,090	62	96	42	
Performance status					
0-1	1,759	100	229	100	1.0
≥2	0	0	0	0	
pT stage					<0.001
1-2	137	7.8	3	1.3	
3	1,042	59	131	57	
4	541	31	95	41	
Unknown	39	2.2	0	0	
N stage					<0.001
0	315	18	76	33	
1	946	54	109	48	
2	457	26	44	19	
NA	39	2.2	0	0	
AJCC disease stage					<0.001
2	314	18	76	33	
3	1,402	80	152	66	
Unknown	42	2.4	0	0	
Stage III Risk Status					<0.001
High	690	39	76	33	
Low	712	40	76	33	
Not applicable (stage II)	357	20	77	33	
Tumour Location					<0.001
Left	1,144	65	36	16	
Right	586	33	193	84	
Unknown	29	1.6	0	0	
Treatment Regimen					0.5
CAPOX	1,241	71	157	69	
FOLFOX	518	29	72	31	
Treatment duration					0.2
12 weeks	876	50	124	54	
24 weeks	883	50	105	46	

pT –pathological tumour (T) stage; MMR – DNA mismatch repair; MMRp – mismatch repair proficient; MMRd – mismatch repair deficient;
*determined by unpaired Student's t-test.
†determined by Fisher exact test (in cases which marker status was determined).

Table S7. Univariable and multivariable analysis of recurrence-free interval in SCOT trial cohort according to clinicopathologic factors and MMR status. Related to Figure 5.

	Univariate					Multivariate				
**Characteristic**	**Cases**	**Events**	**HR[1]**	**95% CI[1]**	***P***	**Cases**	**Events**	**HR[1]**	**95% CI[1]**	***P***
Age	1,959	464				1,942	461			
<70			1.00	—				1.00	—	
>/70			1.03	0.84, 1.26	0.78			0.97	0.79, 1.19	0.8
Gender	1,959	464				1,942	461			
F			1.00	—				1.00	—	
M			0.96	0.79, 1.15	0.63			0.97	0.80, 1.17	0.7
Tumour Stage	1,945	463				1,942	461			
1-2			1.00	—				1.00	—	
3			2.75	1.50, 5.04	0.001			2.76	1.50, 5.07	0.001
4			5.29	2.88, 9.69	<0.001			5.27	2.85, 9.74	<0.001
Nodal Stage	1,943	461				1,942	461			
0			1.00	—				1.00	—	
1			1.41	1.05, 1.87	0.020			1.74	1.29, 2.33	<0.001
2			2.83	2.11, 3.79	<0.001			3.06	2.28, 4.12	<0.001
Sidedness	1,959	464				1,942	461			
Left			1.00	—				1.00	—	
Right			1.41	1.17, 1.69	<0.001			1.30	1.07, 1.58	0.008
Regimen	1,959	464				1,942	461			
Capox			1.00	—				1.00	—	
Folfox			1.11	0.92, 1.35	0.28			1.01	0.83, 1.23	>0.9
Duration	1,959	464				1,942	461			
12 weeks			1.00	—				1.00	—	
24 weeks			0.91	0.75, 1.09	0.28			0.92	0.76, 1.10	0.4
MMR status	1,959	464				1,942	461			
MMRp			1.00	—				1.00	—	
MMRd			0.69	0.50, 0.96	0.029			0.62	0.44, 0.88	0.007

[1]HR = Hazard Ratio, CI = Confidence Interval

Table S8. Biomarker analyses performed and reported in this study. Related to STAR methods.

Analysis	Population	Methods	Reported
RFI by MMR status	Stage II/III CRCs from SCOT trial	Log-rank test, univariable and multivariable adjusted HR	Main text, Figure 5, Table S8
RFI by MMR status	Defined subgroups in SCOT trial	Univariable and multivariable adjusted HR	Figure 5<br>Table S8

RFI – recurrence-free interval; OS – overall survival; HR – hazard ratio. *Full multivariable model included age, gender, location,

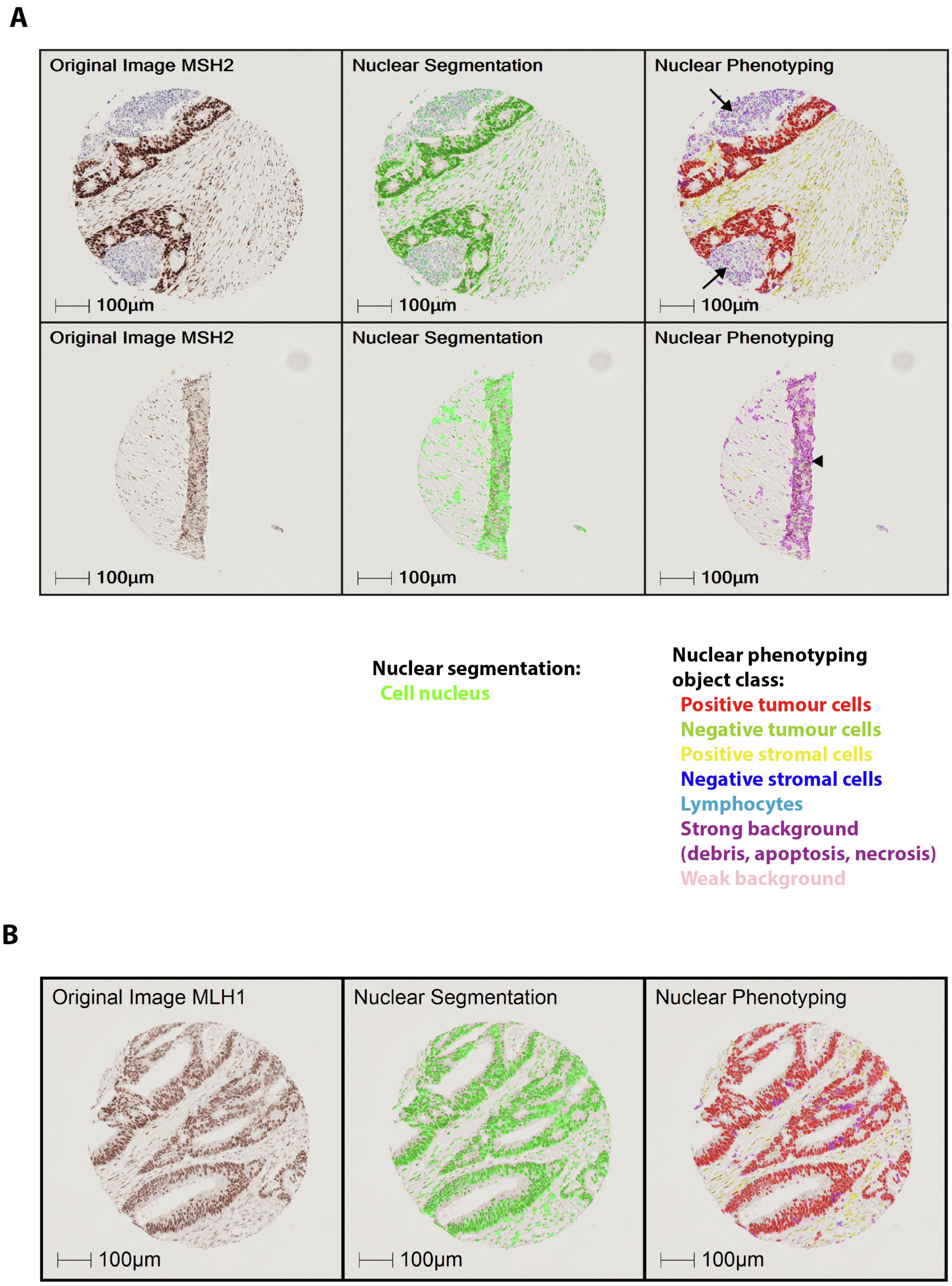

Figure S1. Nuclear segmentation by object class. Related to STAR methods.
(A) Illustrative images of AI-based classification of cell nuclei and other objects on tumor sections following IHC for MMR proteins. Panels show the original IHC stained images (left), nuclear segmentation mark-ups (centre) and nuclear phenotyping by object classification (right). Upper panels show the original image (left), nuclear segmentation (center) and classification results (right) of a representative tissue microarray core with tumor cells, stromal cells and intraglandular debris (black arrows) correctly classified . Lower panels show an exemplary core with a folding artefact (black arrow), with the nuclei in the affected area and adjacent out-of-focus tissue regions correctly classified as strong background (uninformative for analysis) by nuclear phenotyping,. Note that partial transparency in nuclear segmentation masks permits visualisation of DAB staining in MSH2 positive cells.

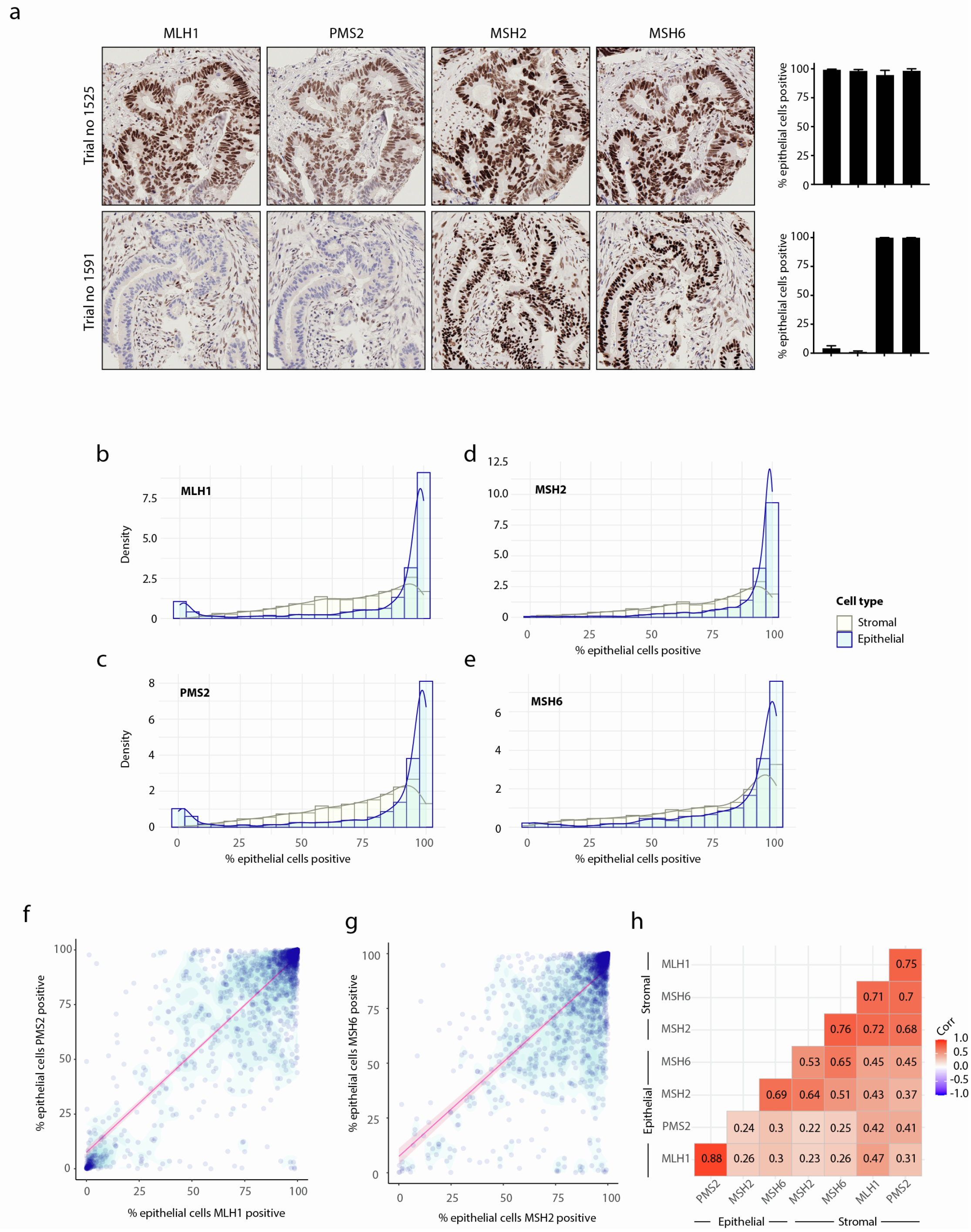

Figure S2. AIMMeR single cell analysis of MMR proteins identifies loss of epithelial expression and expected correlations. Related to Figure 3.
(a) Representative images of immunohistochemistry (IHC) for DNA mismatch repair (MMR) proteins MLH1, PMS2, MSH2 and MSH6 in cases with expression of all proteins (upper row), and loss of MLH1 and PMS2 with retained expression of MSH2 and MSH6 (lower row). Barplots to the right show the percentage of epithelial cells positive for each MMR protein as determined by AI (error bars indicate standard deviation between cores). (b-d) Frequency histograms with overlaid kernel density plots showing proportion of cases by percentage of epithelial and stromal cells positive for MMR proteins MLH1 (b), PMS2 (c), MSH2 (d) and MSH6 (e). (f, g) Scatterplots showing relationship between percentage of epithelial cells expressing dimerization partners MLH1 and PMS2 (f) and MSH2 and MSH6 (g). Regression line represents Spearman rho with 95% confidence intervals. (h) Matrix showing correlation between epithelial and stromal cell positivity for all four MMR proteins ($P<2.2e-16$ all cases). For consistency, plots in (b-h) show results from analysis of 1,988 cases and exclude 27 cases classed as failed on pathologist review; plots from analysis of original 2,015 cases were essentially identical.
.

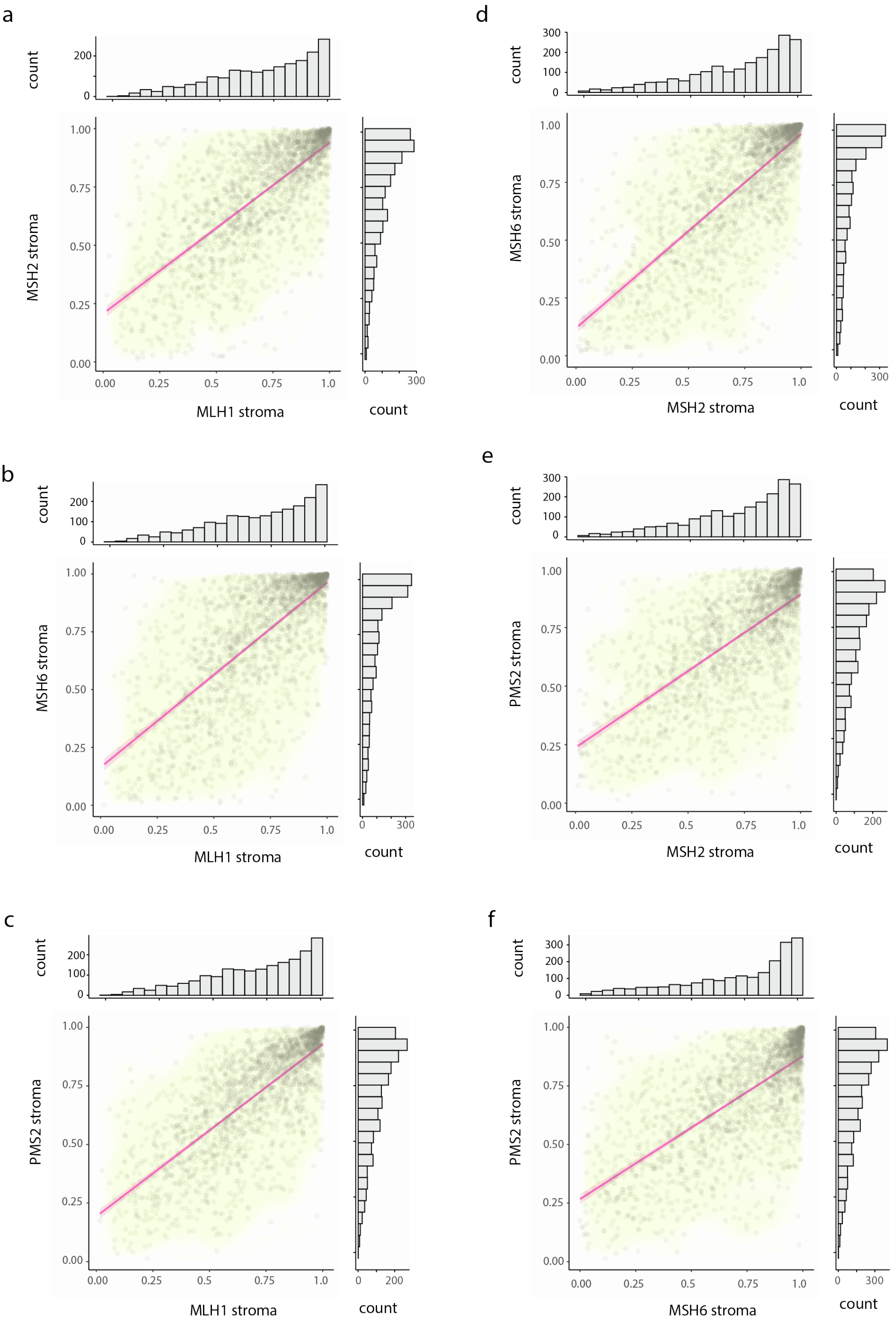

Figure S3. Scatterplots showing correlation between stromal MMR protein expression across cases. Related to Figure 3.

Scatterplots with marginal histograms showing correlation between the proportion of stromal cells positive for individual MMR proteins for: (a) MLH1 and MSH2, (b) MLH1 and MSH6, (c) MLH1 and PMS2; (d) MSH2 and MSH6; (e) MSH2 and PMS2 and: (f) MSH6 and PMS2.

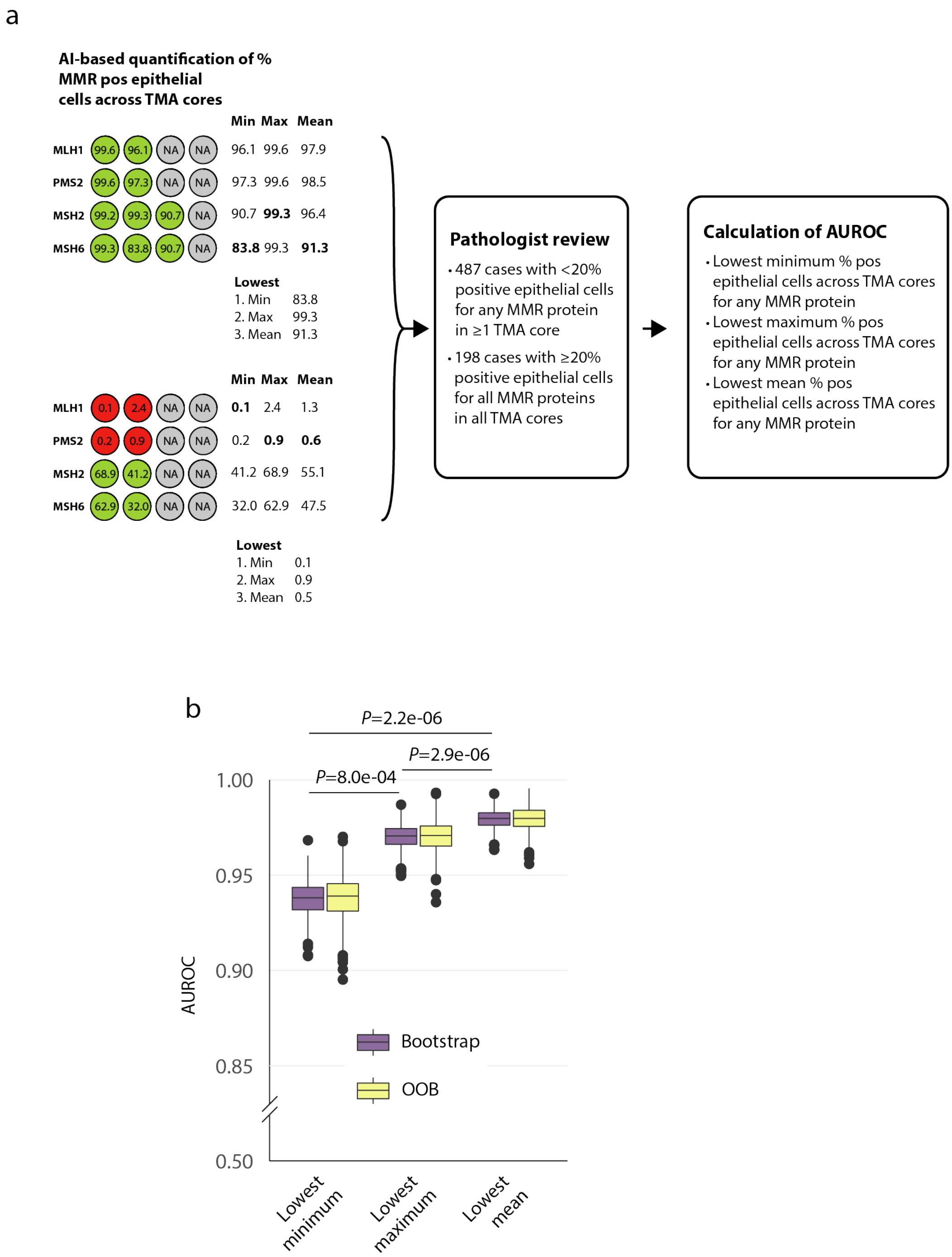

Figure S4. Calculation of AUROC for alternative methods for classification of MMR loss. Related to Figure 3.
(a) Schematic showing study workflow and identification of cases for establishing consensus pathologist ground truth for evaluation of AIMMeR performance. (b) AUROC calculated against consensus pathologist ground truth using alternative metrics based on minimum, maximum and mean percentage of cells positive for individual MMR proteins. Boxplots show median, 25th and 75th percentiles ± 1.5 x interquartile range and outlying points obtained from bootstrap with 1000 resamples and corresponding out of bag (OOB estimates). *P* values were obtained by Mann-Whitney U test.

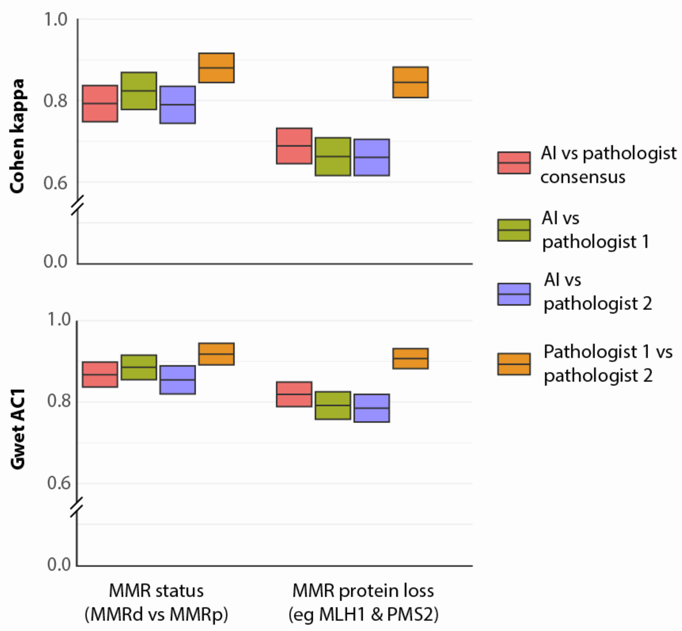

Figure S5. Agreement in classification of MMRd and individual protein loss between AIMMeR, individual and consensus pathologist calls. Related to Figure 3.
Inter-rater reliability measures of agreement between AIMMeR classification of tumour MMR status (left) and combination of MMR protein loss (right) vs individual and consensus pathologist classification. Measures of between-pathologist agreement are shown for comparison. AIMMeR classification uses AIMMeR^{MIN} threshold with maximal Youden index to define MMRd and individual protein loss.

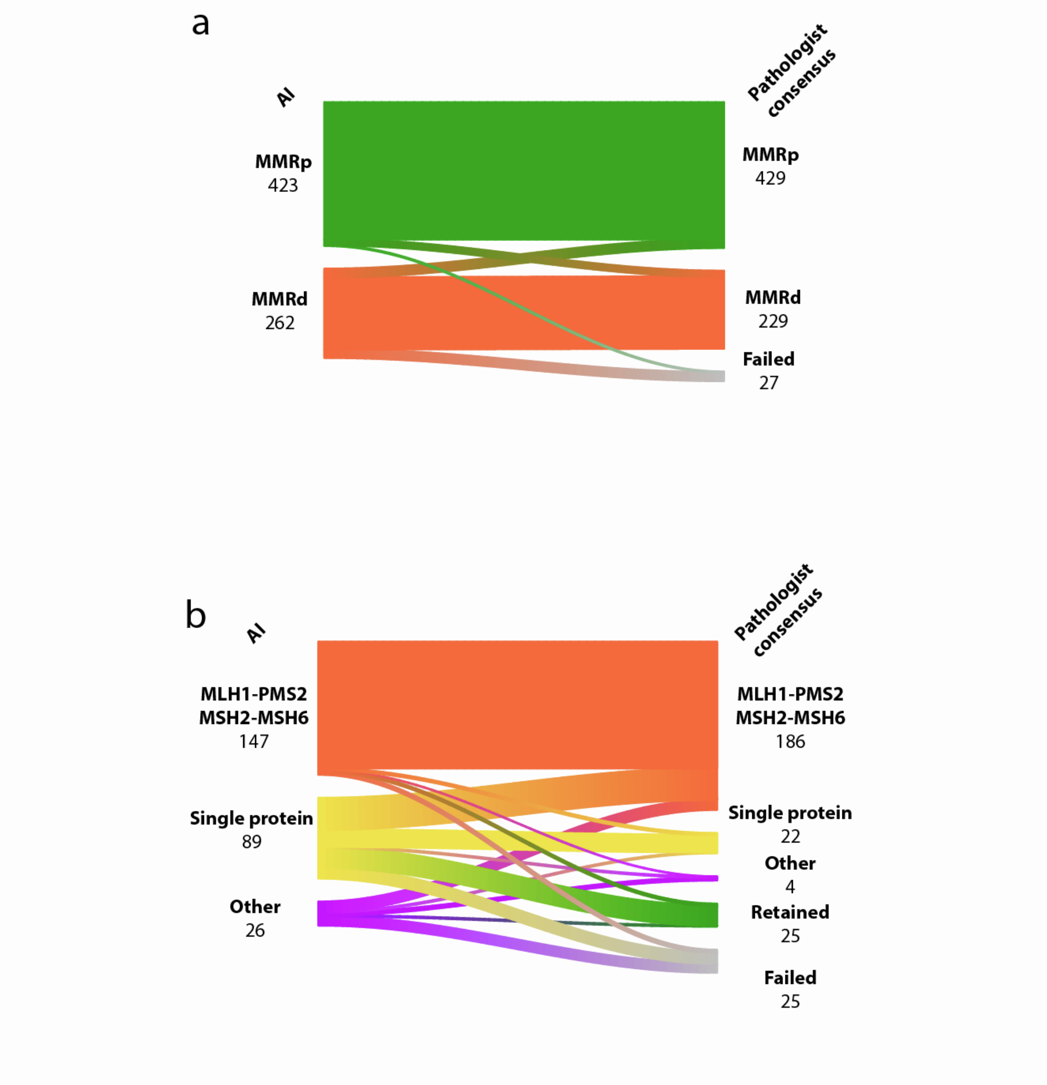

Figure S6. Sankey plots showing relationship between AIMMeR classification of MMR status and individual MMR protein loss. Related to Figure 3.
(a) Plot showing relationship between AIMMeR classification and consensus pathologist review for (a) MMR status and (b) pattern of protein loss. Category of "other" includes MLH1-PMS2 or MSH2-MSH6 loss plus other MMR proteins as well as alternative combinations of loss. AIMMeR calls used AIMMeRMIN threshold with maximal Youden index.

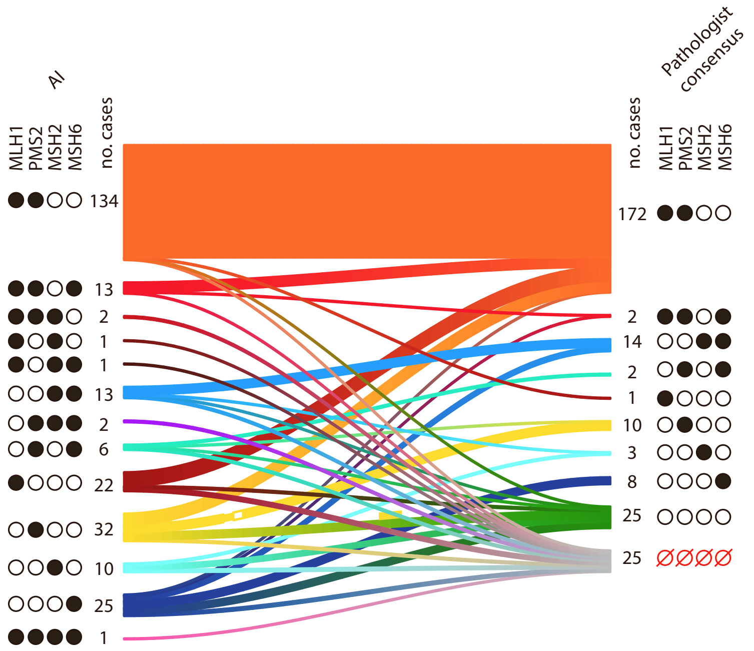

Figure S7. Relationship between AI and consensus pathologist calls for combinations of MMR protein loss. Related to Figure 3.
Sankey plot showing flow between initial AI classification of MMR protein loss using cutpoint of 10.7% positive epithelial cells and final consensus pathologist calls. Proteins lost are indicated by closed circles. Open red circles indicate failed cases.

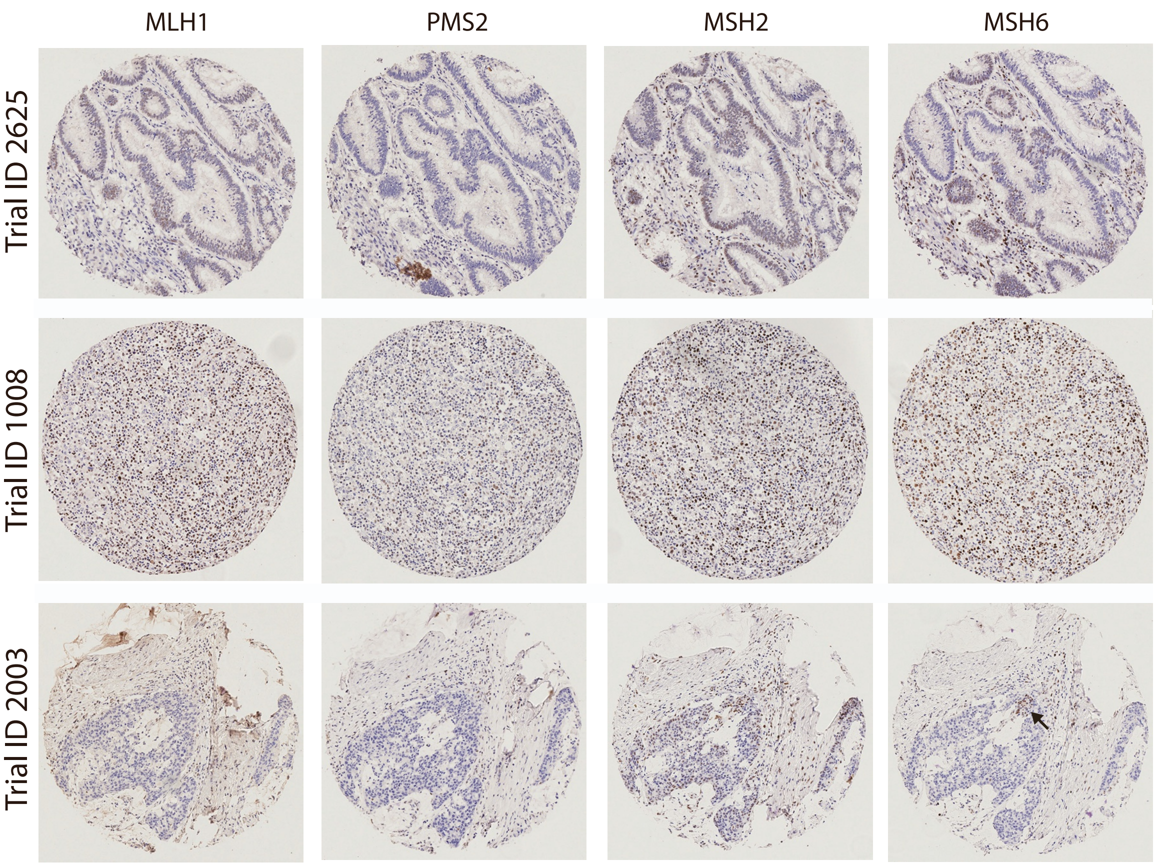

Figure S8. Cases discordant between AI-based and pathologist classification. Related to Figure 3. Representative cases illustrative of causes of AI-pathologist discordance are shown. Upper panel shows failed immunostaining in tumour from participant ID 2625. Immunostain for PMS2 was classified by AI as negative (no positive tumour cells). On pathologist review, this was reclassified to failed staining in view of absence of internal positive controls, and poor quality immunostaining for other MMR proteins. Middle panels show a case misclassified by AI as MMRp as a result of atypical tumour epithelial morphology, in setting of intense lymphocytic infiltrate. On pathologist review, this was reclassified to MMRd with loss of MLH1 and PMS2. Lower panels show a case classified as MMRd by both AI and pathologist review, but discordant for protein loss. AI-based analysis classified this as lacking expression of MLH1, PSM2 and MSH6; the latter owing to MSH6 expression in 2.6% of epithelial cells. Pathologist review identified a small area of retained MSH6 expression (black arrow) in background of loss, leading to reclassification of case as MLH1 and PMS2 deficient with subclonal MSH6 loss.

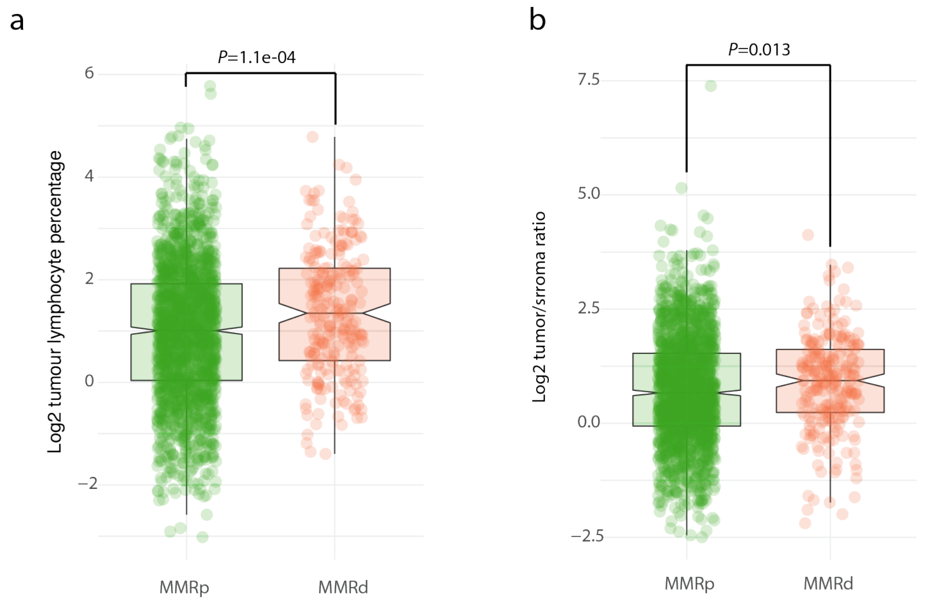

Figure S9. Tumor lymphocytic infiltrate and tumor/stroma ratio by MMR status. Related to Figure 5.
(a) Lymphocytes as percentage of all cells within tumor (determined by single-cell AI-based analysis) according to MMR status. (b) Tumor (malignant epithelial) cell/stroma cell ratio (determined by single-cell AI-based analysis) according MMR status.
